# Yap5 Competes With Hap4 for the Regulation of Iron Homeostasis Genes in the Human Pathogen *Candida glabrata*

**DOI:** 10.3389/fcimb.2021.731988

**Published:** 2021-11-26

**Authors:** Thierry Delaveau, Antonin Thiébaut, Médine Benchouaia, Jawad Merhej, Frédéric Devaux

**Affiliations:** Sorbonne Université, CNRS, Institut de biologie Paris-Seine (IBPS), UMR 7238, Laboratoire de Biologie Computationnelle et Quantitative, Paris, France

**Keywords:** yeast, evolution, stress response, transcription factors, regulation, chromatine immunoprecipitation (ChIP)

## Abstract

The CCAAT-binding complex (CBC) is a conserved heterotrimeric transcription factor which, in fungi, requires additional regulatory subunits to act on transcription. In the pathogenic yeast *Candida glabrata*, CBC has a dual role. Together with the Hap4 regulatory subunit, it activates the expression of genes involved in respiration upon growth with non-fermentable carbon sources, while its association with the Yap5 regulatory subunit is required for the activation of iron tolerance genes in response to iron excess. In the present work, we investigated further the interplay between CBC, Hap4 and Yap5. We showed that Yap5 regulation requires a specific Yap Response Element in the promoter of its target gene *GRX4* and that the presence of Yap5 considerably strengthens the binding of CBC to the promoters of iron tolerance genes. Chromatin immunoprecipitation (ChIP) and transcriptome experiments showed that Hap4 can also bind these promoters but has no impact on the expression of those genes when Yap5 is present. In the absence of Yap5 however, *GRX4* is constitutively regulated by Hap4, similarly to the genes involved in respiration. Our results suggest that the distinction between the two types of CBC targets in *C. glabrata* is mainly due to the dependency of Yap5 for very specific DNA sequences and to the competition between Hap4 and Yap5 at the promoter of the iron tolerance genes.

## Introduction

The CCAAT-binding complex (CBC) is a highly conserved eukaryotic transcription factor, which is composed of three core subunits named NF-YA, NF-YB and NF-YC in mammals and plants, HapB, HapC and HapE in filamentous fungi and Hap2, Hap3 and Hap5 in yeasts (Zhao et al., 2016; Hortschansky et al., 2017; Li et al., 2018; Mao and Chen, 2019). In fungi, the CBC is constitutively bound to its target genes and requires a fourth regulatory subunit to control transcription in a condition-specific manner. For instance, in the model yeast *Saccharomyces cerevisiae*, CBC interacts with the Hap4 protein to positively control the expression of “respiratory genes” involved in respiration, TCA cycle, gluconeogenesis, heme biosynthesis and mitochondria biogenesis (Bolotin-Fukuhara, 2017). This allows this yeast to induce respiration specifically upon glucose deprivation or during growth in non-fermentable carbon sources such as glycerol. Hence, Hap4 is subjected to glucose catabolic repression and its expression level is under complex transcriptional and post-translational regulations (Forsburg and Guarente, 1989; Bouchez et al., 2020). Hap4 interacts with the Hap5 core CBC subunit through a short 16 amino acids motif, called the Hap4-Like CBC binding domain (Hap4L), which is located at the N-terminus of the protein (McNabb and Pinto, 2005; Bolotin-Fukuhara, 2017). Of note, Hap4 does not have a specific DNA binding domain and its target selection is thought to occur indirectly through its association with the CBC. Yet, the non-specific interaction of Hap4 with the DNA surrounding the CCAAT box was shown to be required for its proper association with the CBC (McNabb and Pinto, 2005).

Clear orthologues of Hap4 have been found only in Hemiascomycetous yeasts (Petryk et al., 2014). However, in many other fungal species, including numerous pathogens, another CBC regulatory subunit, named HapX, has been identified and described as a major regulator of iron homeostasis in these species (Misslinger et al., 2021). HapX only shares with Hap4 the Hap4L domain, allowing its association with the CBC. Upon iron deprivation, the HapX-CBC complex acts as a repressor of genes involved in iron-dependent pathways, i.e. genes encoding iron-sulfur cluster and heme containing proteins or involved in the biogenesis and assembly of these two prosthetic groups, and genes encoding transporters responsible for vacuolar sequestration of iron (Hortschansky et al., 2007; Jung et al., 2010; Singh et al., 2011; Lopez-Berges et al., 2012; Kröber et al., 2016; Wang et al., 2019; Do et al., 2020). In the human and plant pathogens *Aspergillus fumigatus, Fusarium oxysporum* and *Verticillium Dahliae*, HapX is also responsible for the induction of some of these “iron consuming genes” in high iron conditions, therefore playing a key role in iron tolerance (Gsaller et al., 2014; Wang et al., 2018). However, this latter role of HapX seems to be less conserved across fungi than its function as a repressor upon iron deprivation (Skrahina et al., 2017).

HapX and its orthologues are transcriptionally induced by iron starvation and sense iron through conserved cysteine rich domains (CRD) in their C-terminal part (Hortschansky et al., 2007; Schrettl et al., 2010; Hsu et al., 2011; Singh et al., 2011; Lopez-Berges et al., 2012; Gsaller et al., 2014; Rietzschel et al., 2015; Kröber et al., 2016; Do et al., 2020). Importantly, HapX also contains a basic leucine zipper (bZIP) domain and belongs to the Yeast AP-1 (YAP) family of specific DNA binding transcriptional regulators (Tanaka et al., 2002; Singh et al., 2011; Rodrigues-Pousada et al., 2019). However, in contrast with the other members of this family, HapX does not specifically recognize Yap Response Elements (YRE, canonical sequence TTA(C/G)TAA) in its target promoters. The main consensus sequence found in HapX target promoters is the CCAAT box (CCAAT in most species, C(C/G)AAT in *C. neoformans*) which is bound by the CBC (Chen et al., 2011; Gsaller et al., 2014; Do et al., 2020; Furukawa et al., 2020). Still, the bZIP domains of HapX in *A. fumigatus* and its orthologue in the pathogenic yeast *Candida albicans* (named Hap43) are essential for growth in low iron conditions, optimal DNA binding and optimal gene expression regulation (Singh et al., 2011; Srivastav et al., 2018; Furukawa et al., 2020). Moreover, it was shown in *A. fumigatus* and *A. nidulans* that direct DNA binding of HapX to sequences downstream of the CCAAT box, although with a relatively low specificity and a high variability in the recognized DNA motifs, contributes to target selection and binding affinity of the CBC-HapX complex (Gsaller et al., 2014; Hortschansky et al., 2015; Furukawa et al., 2020). Consistent with the key role of iron in microbial proliferation and nutritional immunity of the host, HapX orthologues have been shown to be important virulence factors in several humans, insect and plant fungal pathogens (Jung et al., 2010; Schrettl et al., 2010; Hsu et al., 2011; Lopez-Berges et al., 2012; Peng et al., 2020).

*Candida glabrata* is an opportunistic human pathogen, which is phylogenetically closely related to the model yeast *S. cerevisiae* (Gabaldon and Fairhead, 2019). As such, its genome encodes a clear orthologue of Hap4 but earlier studies did not identify obvious *C. glabrata* equivalents of HapX (Petryk et al., 2014), which was consistent with the fact that the regulatory networks controlling iron homeostasis have significantly diverged between *Saccharomycetaceae* yeasts (e.g. *S. cerevisiae* and *C. glabrata*) and other fungal species (e.g. *C. albicans*, *A. nidulans*, *C. neoformans*, *S. pombe*, etc.) (Gerwien et al., 2018). However, by combining high-throughput chromatin immunoprecipitation experiments (ChIP-seq) with transcriptomics and co-immunoprecipitation analyses of the Hap5 CBC core subunit, we previously showed that the *C. glabrata* CBC has a dual role in respiration and iron homeostasis (Thiébaut et al., 2017). The *C. glabrata* CBC is required for the expression of respiratory genes and it is essential for their strong induction upon growth with non-fermentable carbon sources. As in *S. cerevisiae*, this function seems to require Hap4, since the deletion of *HAP4* severely reduced the expression of the *ATP2* gene, encoding the beta subunit of the mitochondrial ATP synthase and taken as a model for respiratory genes in *C. glabrata* (Thiébaut et al., 2017). In addition to this “*S. cerevisiae*-like” function, and more unexpectedly, the *C. glabrata* CBC was found to bind the promoters of genes known in *S. cerevisiae* to be required for adaptation to toxic iron concentrations (Thiébaut et al., 2017). More specifically, these iron related targets of *C. glabrata* CBC include the vacuolar iron transporter encoded by *CCC1*, the glutaredoxin involved in iron signaling and iron sulfur cluster assembly encoded by *GRX4*, the iron-containing enzymes aconitase (encoded by *ACO1*), sodium dehydrogenase (encoded by *SDH2*), glutamate synthase (encoded by *GLT1*) and wybutosine synthase (encoded by *TYW1*), the essential iron-sulfur cluster containing termination factor encoded by *RLI1*, a protein involved in the maturation of iron-sulfur cluster mitochondrial proteins (encoded by *ISA1*) and the porphobilinogen deaminase (encoded by *HEM3*) which catalyzes a key step in the biosynthesis of heme. The deletion of *HAP5* in *C. glabrata* had few impact on the expression of these “iron tolerance genes” in optimal growth conditions, but it completely abolished their induction in response to toxic concentrations of iron. However, in contrast with the role of the CBC in other fungal pathogens, Hap5 had a very limited impact on gene expression in iron starvation conditions *in C. glabrata* (Thiébaut et al., 2017).

In *S. cerevisiae*, the high iron induction of iron tolerance genes had previously been shown to be dependent on Yap5 (Li et al., 2008; Li et al., 2011; Pimentel et al., 2012). Yap5 is a bZip transcription factor of the YAP family (Fernandes et al., 1997), which constitutively and specifically binds a canonical YRE motif in the promoter sequence of its target genes (Li et al., 2008; Tan et al., 2008; Pimentel et al., 2012). Like HapX, Yap5 senses iron excess by conserved CRDs at its C-terminus (Rietzschel et al., 2015). This interaction changes Yap5 conformation, turning it into a strong transcriptional activator (Li et al., 2008; Rietzschel et al., 2015). Unlike HapX, Yap5 has very few impact on gene expression upon iron deprivation (Pimentel et al., 2012; Merhej et al., 2016).

The role of Yap5 in iron tolerance was shown to be well conserved in *C. glabrata* and, until the discovery that Hap5 was also required for adaption to high iron in this species, Yap5 was thought to act on his own in this process (Merhej et al., 2016). However, careful examination of Yap5 sequence in *C. glabrata* revealed that it actually exhibits a truncated Hap4L domain lacking the last four C-terminal amino acids of the canonical motif (Merhej et al., 2015). This domain was shown to be required for Yap5 binding to its target promoters and to mediate interaction of Yap5 with Hap5 (Thiébaut et al., 2017). Moreover, the presence of Hap5 was absolutely required for Yap5 binding to its targets, explaining why the CBC is essential to the high iron response in *C. glabrata* (Thiébaut et al., 2017). These findings revealed that, although their roles in the regulation of iron homeostasis considerably diverged, Yap5 and HapX are actually homologous proteins harboring many similarities in their functioning, and that they probably derived from a common ancestor.

The two roles of CBC in *C. glabrata* were found to be independent: Hap4 was shown to be completely dispensable for the high iron induction of *GRX4*, used as a model for iron tolerance genes, and reciprocally, the deletion of *YAP5* had no impact on the expression of *ATP2*, used as a model for respiratory genes (Thiébaut et al., 2017). Interestingly enough, the sets of iron consuming genes regulated by HapX in *A. fumigatus*, *C. neoformans* or *C. albicans* includes both orthologues of the *C. glabrata* respiratory genes supposed to be regulated by Hap4 (e.g. *CYB2*, *CYC1*, *RIP1*, *SDH1*, *CYT1*) and orthologues of the *C. glabrata* iron tolerance genes controlled by Yap5 (e.g. *CCC1*, *GLT1*, *HEM3*, *ISA1*, *RLI1*, *TYW1*, *ACO1*, *SDH2*) (Chen et al., 2011; Hsu et al., 2011; Singh et al., 2011; Gsaller et al., 2014; Do et al., 2020; Furukawa et al., 2020), suggesting that the *C. glabrata* CBC regulatory network might have evolved by rewiring the roles of the Hap4 and Yap5 CBC regulatory subunits from an ancestral HapX-like situation.

Still, the co-existence of these two active CBC regulatory subunits in *C. glabrata* raises an important question. Since Hap4 and Yap5 both have the ability to bind CBC, how do they distinguish between the CBC bound to respiratory genes and the CBC bound to iron tolerance genes? In other terms, what are the underlying mechanisms rendering the two pathways independent from one another and preventing functional interferences between them? In the present work, we addressed this question by combining directed mutagenesis, ChIP and gene expression analyses in various mutants. We showed that the regulation of iron tolerance genes by Yap5 is dependent, not only on its interaction with CBC, but also on its specific binding to YRE, thus restricting its action to promoters in which a CCAAT and a YRE are present close to one another. The presence of Yap5 at these promoters considerably strengthened the binding of CBC on these genes, therefore suggesting a strong synergy between the CCAAT-CBC-Yap5-YRE interactions. Additionally, ChIP-seq experiments identified significant Hap4 binding at respiratory and iron tolerance genes, indicating that Hap4 apparently does not distinguish between these two gene categories. However, in contrast to respiratory genes, Hap4 binding at iron tolerance genes was apparently much lower than Hap5 binding at those same promoters, suggesting that Hap4 is not the preferred partner of CBC on these genes. Moreover, transcriptomic analyses confirmed that Hap4 has no role in the high iron response, while it is required for proper expression of respiratory genes. Analyses of *GRX4* expression levels in a *HAP4* and *YAP5* double deletion mutant indicated that the competition with Yap5 prevents Hap4 to act on the transcription of iron tolerance genes. More precisely, in the absence of Yap5, *GRX4* showed a residual expression level which was independent of iron and dependent on Hap4, hence harboring a “respiratory gene-like” pattern of expression.

## Material and Methods

### Strains and Primers

The list of the primers and strains used in this study is available in **Supplementary Files S1, S2**, respectively. All the simple deletion mutant strains used in this study were obtained from existing collections (Schwarzmuller et al., 2014) and were verified by PCR before use, except for the *Δyap5* strain which was re-constructed for this study in the same genetic background than the Hap5 and Hap4 mutants (see below). The genomic Hap5 myc-tagged strain was published previously (Thiébaut et al., 2017). The genomic myc-tagging of Hap4 was performed as described previously (Merhej et al., 2015). Briefly, myc-tagging cassette was PCR amplified from the M. Longtine’s plasmids with oligonucleotides containing homology sequences flanking the desired genomic insertion point in 5 ′ (Longtine et al., 1998). At least 10 micrograms of purified PCR product was used to transform wild type cells (HTL background, Schwarzmuller et al., 2014) using a standard yeast transformation protocol (Merhej et al., 2015). Genotyping of the clones growing on selective media was done by PCR. The correct myc-tagging of Hap4 was verified by sequencing of the gene and western blot (Thiébaut et al., 2017). The deletion of *YAP5* in the wild type, *Δhap4*, Hap5-myc or Hap4-myc strains was performed as previously described (Merhej et al., 2015). The actual deletion at *YAP5* locus and the absence of duplication of *YAP5* elsewhere in the genome were controlled by PCR.

To mutate the *GRX4* promoter, we amplified by PCR the 500 base pairs upstream of *GRX4* translation start codon and cloned the PCR product in the pSG plasmid upstream to the *LacZ* reporter gene contained in this plasmid, using SmaI and NotI restriction enzymes. The pSG plasmid was obtained from the pZLG plasmid (Garcia et al., 2010) by replacing the *S. cerevisiae* Autonomous Replicating Sequence (ARS) and the *URA3* marker by the *C. glabrata* ARS and the *HIS3* marker from the pGRB2-1 plasmid (Frieman et al., 2002), using KpnI and XhoI restriction enzymes. We then used the Quick change site-directed mutagenesis kit from Agilent, following the recommendations of the supplier, to introduce mutations in the CCAAT or the YRE motif in the wild type version of the *GRX4* promoter. The mutant and wild type plasmids were controlled by sequencing. After transformation in *C. glabrata* using the “one-step” yeast transformation protocol (Merhej et al., 2015), the presence of the desired mutations and the absence of additional variations were controlled by Sanger sequencing.

### Yeast Cultures and Growth Conditions

All cultures were grown in a rotative shaker at 30°C. The standard growth media was YPD (Glucose 2%, yeast extract 1%, Bactopeptone 1%). For growth in non-fermentable conditions, 2% glucose was replaced by 2% glycerol (YPGly). For iron excess conditions, the cells were grown in CSM (2% glucose, 0.67% Yeast Nitrogen base, 0.08% Complete Synthetic Media (MP Bio)) and 2 mM iron sulfate were added at the desired optical density (OD). The cells were then exposed to the stress for one hour.

### Chromatin Immunoprecipitation and High-Throughput Sequencing (ChIP-*Seq*)

For ChIP, myc-tagged strains were grown in YPGly until exponential phase (OD = 0.8). Cross-linking of the cells and ChIP-seq were performed as described previously (Lelandais et al., 2016). The parental HTL (untagged strain) was grown and processed the same way to provide the mock-IP samples. Sequencing of the IPs, Input DNAs and mock IPs samples and primary data analyses (quality controls and mapping of the reads) were performed as described previously (Lelandais et al., 2016). Peak calling was performed with the bpeaksApp software (Denecker and Lelandais, 2018), using both the Input DNA and the mock IP as references (Merhej et al, 2014; Lelandais et al., 2016). For peak calling, the bpeaks parameters were T1 = 3, T2 = 3, T3 = 1.5, T4 = 0.6 for Hap4 and T1 = 2, T2 = 4, T3 = 0.9, T4 = 0.8 for Hap5. The selected peaks were then manually checked on a genome browser (Thorvaldsdottir et al., 2013) to discard artefactual peaks (e.g., peaks centered on a tRNA locus, peaks perfectly overlapping a highly expressed ORF) which would have escaped the bpeaks filter. Peaks located outside of a promoter region (i.e. between convergent ORF or inside ORFs) were also discarded from the final list presented in **Supplementary Table T1**.

For ChIP-Q-PCR experiments, the ChIP was performed using the Dyagenode Ideal kit, following the supplier recommendations.

### Network Building

The ChIP peaks were assigned to genes as described previously (Merhej et al., 2016). When a peak was located in a divergent promoter (i.e., an intergenic region between two divergent genes) the two genes were fused in one target in the network named “gene 1/gene 2” (**Supplementary Table T1**). The network was represented using Cytoscape (Shannon et al., 2003).

### DNA Motif Enrichment Analyses and Promoter Sequences Analyses

To analyze motif enrichment in Hap4 and Hap5 ChIP-seq peaks, DNA sequences of ChIP peaks were retrieved from their genomic locations using the “getfasta” function from the BEDTOOLS suite (Quinlan and Hall, 2010). These genomic sequences were used as inputs for the peak-motif tool to search for enrichment of regulatory motifs (Thomas-Chollier et al., 2012).

To check the presence of a bipartite motif in the promoter sequences of *GRX4*, *CCC1*, *ISA1*, *TYW1*, *HEM3* and *RLI1* orthologues in different yeast species, the 800 base pairs upstream of the ATG of the corresponding genes were downloaded from GRYC (http://gryc.inra.fr/) or from the NCBI (for species not supported by GRYC) (https://www.ncbi.nlm.nih.gov/genome/). For *GLT1*, the 2000 base pairs upstream of the ATG was used, since the Hap5/Yap5 ChIP peak in *GLT1* promoter was located more than 1 kb upstream of the ATG (Thiebaut et al., 2017). We first searched on the two strands for CCAAT box or its known variations (CGAAT and CCATT) and then looked for a YRE (TTACTAA, TTAGTAA, TGAGTAA or TGACTAA) in the 50 bp upstream or downstream from the CCAAT. The seqlogo of the bipartite motif from *Saccharomycetaceae* orthologues of *GRX4* was generated using WebLogo (Crooks et al., 2004).

### Transcriptome Analyses

For the glycerol condition, knock-out and wild type strains were grown in 50 mL of YPD until exponential phase (OD = 0.8), then washed two times with sterile water, resuspended in 50 mL of YPGly. After one hour, 15 mL of each cell cultures were flash-frozen in two volumes of cold ethanol and collected by centrifugation. For high iron experiments, knock-out and wild type strains were grown in 50 mL of CSM until exponential phase (OD = 0.8) and then 2 mM of iron sulfate were added. After one hour, 15 mL of each cell cultures were flash-frozen in two volumes of cold ethanol and collected by centrifugation. Total RNA was extracted, quality controlled and quantified as described previously (Merhej et al., 2015). One microgram of total RNA was used for fluorescent cDNA synthesis according to the amino-allyl protocol. The cDNAs were labeled with Cy3 and Cy5 and hybridization was performed as previously described (Merhej et al., 2015). Three biologically independent experiments were performed for each condition, using dye switch. We used custom *C. glabrata* Agilent arrays in an 8 × 60 k format (array express accession number: A-MEXP-2402). After overnight hybridization and washing, the slides were scanned using a 2- micron Agilent microarray scanner. The images were analyzed using the feature extraction software (Agilent technologies) and normalized using global LOESS (Lemoine et al., 2006). The mean of the biological triplicates was calculated. Hierarchical clustering was performed using MeV with Euclidian distance, optimization of gene leaf order and average linkage (Saeed et al., 2003).

### Real Time Quantitative PCR (qPCR) Analyses

For RNA extraction, cDNA synthesis and qPCR, cells were grown, collected and total RNAs were extracted as described in section ***Transcriptome Analyses***. For each sample, 10 μg of the total RNA were DNAse treated using Turbo DNA-free kit (Ambion). After DNAse treatment, 0.2 μg of total RNA were used to perform cDNA synthesis using Superscript II Reverse Transcriptase according to the manufacturer’s instructions (Invitrogen). The resulting cDNA were diluted to three different concentrations (1:10, 1:20 and 1:40). qPCR reactions were performed on a C1000 TM Thermalcycler (Bio-rad) with a 2X SYBR Green master mix (Promega). The qPCR reaction mixture contained 0.5 μM of each primer and 4 μL of one of the three dilutions of the cDNA. These dilutions served as technical triplicate for each sample. The primers used are listed in **Supplementary File S1**. The relative expression of a gene was calculated as the difference in the abundance between the transcripts of this gene compared to the transcripts of the *ACT1* gene, used as a reference, based on the ΔCt method. Finally, the expression values were normalized with the expression of the studied gene in the wild type strain grown in glucose set to 1.

For Chromatin-immunoprecipitation followed by qPCR (ChIP-qPCR), three serial dilutions (1:4, 1:8, 1:16) of immunoprecipitated samples were simultaneously processed together with Input samples used for normalization. The primers used are listed in **Supplementary File S1**. The enrichment of the *ACT1* promoter was used as an endogenous control. qPCR was performed as described above. The relative enrichment of a specific locus in the immunoprecipitated DNA relatively to the Input DNA and to the *ACT1* promoter enrichment was determined using the ΔΔCt method.

### Data Availability

The ChIP-seq and microarray data can be downloaded from the Array express database (accession numbers: E-MTAB-10675 and E-MTAB-10544).

## Results

### A Conserved Bipartite CCAAT-YRE DNA Motif Is Required for the High Iron Response in *C. glabrata*

In *S. cerevisiae*, the regulation of the high iron response by Yap5 requires the presence of one or several YRE in the promoters of its target genes (Li et al., 2008; Tan et al., 2008; Pimentel et al., 2012). In *C. glabrata*, a canonical YRE TTA(C/G)TAA was found to be the most significantly enriched DNA motif in the Yap5 target promoters, but the importance of this sequence was not experimentally examined (Merhej et al., 2016). So, an obvious hypothesis to explain the specificity of Yap5 for the promoters of iron tolerance genes in *C. glabrata* would be that it requires both interaction with the CBC, as previously established (Thiébaut et al., 2017), and binding to a YRE to control the expression of its targets.

**Figure 1:**
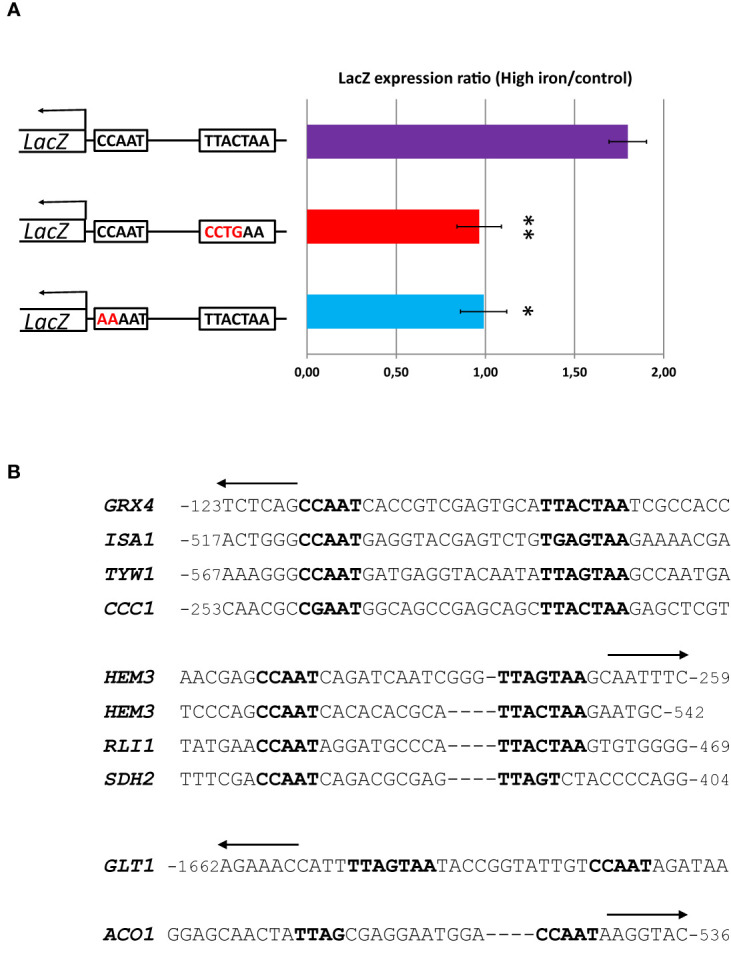
A bipartite CCAAT-YRE motif is required for the high iron response of *GRX4* and probably of most Yap5 targets. **(A)** RT-qPCR analyses of the high iron response (glucose + 2 mM FeSO_4_) of a *LacZ* reporter driven by different versions of the *GRX4* reporter. First line: wild type version. Second line: Mutant version in which the YRE (TTACTAA) of the wild type version has been changed into CCTGAA. Third line: Mutant version in which the CCAAT of the wild type version has been changed into AAAAT. The values represent the expression levels of the *LacZ* reporter relative to *ACT1* (used as an internal control) and to the wild type grown in glucose with normal iron concentration. The experiments were performed two times on biologically independent samples. Error bars hence represent the standard error of the mean. The results of a t-test comparing the results of the wild type version with the YRE mutation and the CCAAT mutation, respectively, are symbolized as follows: **p < 0.05, *p < 0.1. **(B)** Multiple alignments of the CCAAT-YRE bipartite motifs in the promoters of the 9 Yap5 targets. All sequences are oriented 5’-3’ from left to right. The arrows on top indicate the sense of transcription of the corresponding genes, i.e. an arrow oriented to the left indicates that the CCAAT-YRE motif is on the minus (non coding) strand, whereas an arrow oriented to the right indicates that the CCAAT-YRE motif is on the plus (coding) strand. The position relative to the ATG of the gene is indicated. The sequences were arbitrarily aligned on the CCAAT box and on the YRE. Gaps were added in sequences where necessary, to visualize the difference in spacing between the CCAAT and the YRE in the different promoters.

To assess experimentally the importance of the YRE for the high iron response mediated by Yap5 in *C. glabrata*, we cloned the reporter gene *LacZ* under the control of the promoter region of *GRX4*, one of the main targets of Yap5, in a centromeric plasmid. We performed directed mutagenesis to invalidate either the CCAAT box or the YRE in this promoter. We measured the impact of these mutations on the high iron induction of the *LacZ* reporter gene by RT-qPCR. We observed that mutations of either the CCAAT box or the YRE abolished the high iron response of the promoter, as compared to the wild type version (**Figure 1A**).

Hence, the CCAAT box and the YRE seems to be both necessary for the high iron response of *GRX4*. Consistent with this result, a CCAAT or a CGAAT box and a YRE were found in close proximity in 7 out of 9 Yap5 target promoters (**Figure 1B**). More precisely, a canonical YRE (TTACTAA, TTAGTAA or TGAGTAA) was found in close proximity, either downstream (*GRX4*, *ISA1*, *TYW1*, *CCC1*, *HEM3* and *RLI1*) or upstream (*GLT1*), of the CCAAT box. The spacing between the two motifs varied in a short range of 10 (*RLI1* and second binding site in *HEM3*), 12 (*GLT1*), 13 (*HEM3* first binding site) and 14 base pairs (*GRX4*, *ISA1*, *CCC1* and *TYW1*). As reported previously, this configuration is very rare in *C. glabrata*, being present in only 28 promoters over the whole genome (including the 7 targets mentioned just above) (Thiébaut et al., 2017).

The two exceptions among Yap5 targets are *SDH2* and *ACO1*, which only showed half YRE sites located 10 bp downstream and 11 bp upstream of the CCAAT box, respectively (**Figure 1B**). Noteworthy, *SDH2* and *ACO1* are the sole Yap5 targets to also belong to the respiratory gene group, which expression is regulated by Hap4 (see next sections).

**Figure 2:**
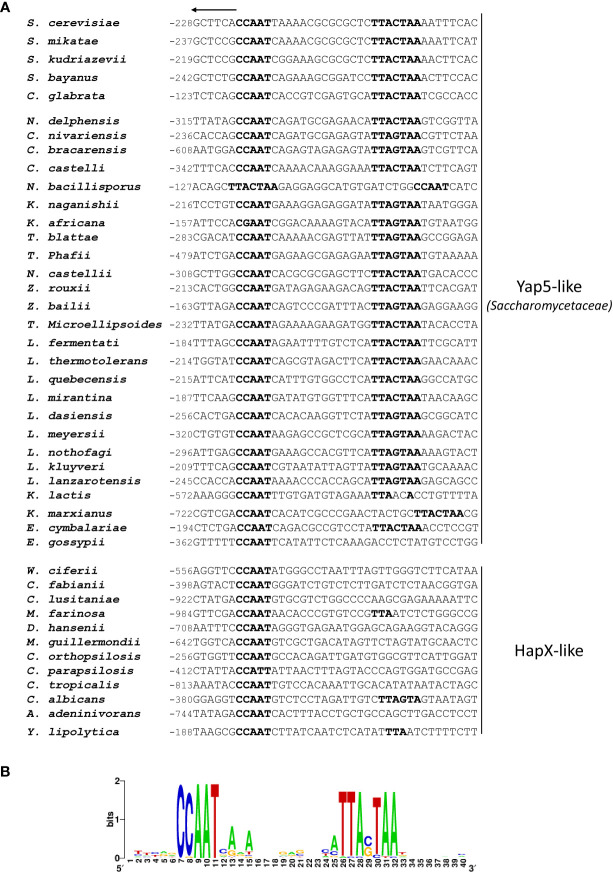
Conservation of the CCAAT-YRE bipartite motifs in *GRX4* orthologues from 43 Hemiascomycetes yeast species. **(A)** Alignment of the CCAAT-YRE bipartite motifs. The sequences were arbitrarily aligned based on the CCAAT box, except for *N. bacillisporus* in which the YRE was located upstream of the CCAAT. The CCAAT and YRE are represented in bold. All sequences are oriented 5’-3’ from left to right. The arrow on top indicate the sense of transcription of *GRX4*, i.e. the arrow oriented to the left indicates that the CCAAT-YRE motif is on the minus (non coding) strand. The position relative to the ATG of the gene is indicated. **(B)** Seqlogo of the CCAAT-YRE alignments in the sequences from the *Saccharomycetaceae* species (“Yap5-like” species) presented in panel **(A)**, excluding *N. bacillisporus*.

Additionally, we studied the conservation of this bipartite CCAAT-YRE motif in the promoter of the *GRX4* orthologues in 43 yeast species (**Figure 2**). Based on their type of Hap4L-bZip protein, budding yeasts can be roughly split in two groups: *Saccharomycetaceae* (e.g. *S. cerevisiae*, *C. glabrata*, *Lachancea kluiveri*, ….) in which the Hap4L-bZip protein is more similar to Yap5 and is involved in the induction of iron tolerance genes upon iron excess (Merhej et al., 2015; Merhej et al., 2016) and all the other species (e.g. *C. albicans*, *Debaryomyces hansenii*, *Yarrowia lipolytica*,…) in which the Hap4L-bZip protein is more similar to HapX and is involved in the repression of iron consuming genes upon iron starvation (Singh et al., 2011; Merhej et al., 2015). Except for a few exceptions (i.e. *N. bacillisporus*, *K. lactis*, *K. marxianus*, *E. gossypii*), we observed an impressive conservation of the CCAAT-YRE motif in the “Yap5-containing” species, at the level of the motifs sequences and at the level of the 14 bp spacing between the two motifs (**Figures 2A, B**). This signal was completely lost in “HapX-containing” species (**Figure 2A**). This conservation pattern strongly suggests that this bipartite motif is under selection in *Saccharomycetaceae* and supports the hypothesis that it is important for the regulation of *GRX4* by Yap5. Similar conservation patterns were observed for the CCAAT-YRE motifs in the *CCC1*, *ISA1* and *TYW1* promoters, with some variations (for instance the motif was apparently lost in *ISA1* orthologues in the *Zygosaccharomyces* clade) (**Supplementary Files S3–S5**). The CCAAT-YRE in *HEM3* was only present in some post-WGD species (e.g. *Saccharomyces* and *Nakaseomyces* clades) with frequent variations in the spacing between the CCAAT and the YRE (**Supplementary File S6**), while the CCAAT-YRE motif in *RLI1* was mostly restricted to the three *Nakaseomyces* species which are close to *C. glabrata* (**Supplementary File S7**). In contrast, the unusual YRE-CCAAT motif in *GLT1* was not conserved, even in species very closely related to *C. glabrata* (**Supplementary File S8**). Of note, this YRE-CCAAT orientation in *GLT1* is also in contradiction with the 3D structure of the *A. nidulans* CBC bound to the CCAAT box, which showed that its Hap4L recruitment domain is positioned downstream of the CCAAT box (Huber et al., 2012). These two observations (lack of conservation and discrepancy with previous structural data) make the actual functioning of the YRE-CCAAT in *GLT1* questionable.

As a conclusion to this section, a conserved CCAAT-YRE seems to be required for Yap5 regulation of most of its target genes, which may partly explain the specificity of targets of the Yap5-CBC complex in *C. glabrata*.

### Hap4 Binds Both Respiratory Genes and High Iron Tolerance Genes, but With a Quantitatively Different Pattern as Compared to Hap5

To get a genome-wide view of Hap4 DNA binding pattern, we performed ChIP-seq experiments using a *C. glabrata* strain in which *HAP4* had been tagged with 13 myc epitopes at its chromosomic location. These experiments were conducted on cells grown in glycerol media, as the expression level and activity of Hap4 had been shown to be maximal in non-fermentable carbon sources conditions in *S. cerevisiae* (Forsburg and Guarente, 1989; Bouchez et al., 2020). In parallel, we also performed ChIP-seq experiments on a Hap5 myc-tagged strain grown in the exact same conditions, to get comparable data of DNA binding patterns between Hap4 and the CBC.

**Figure 3:**
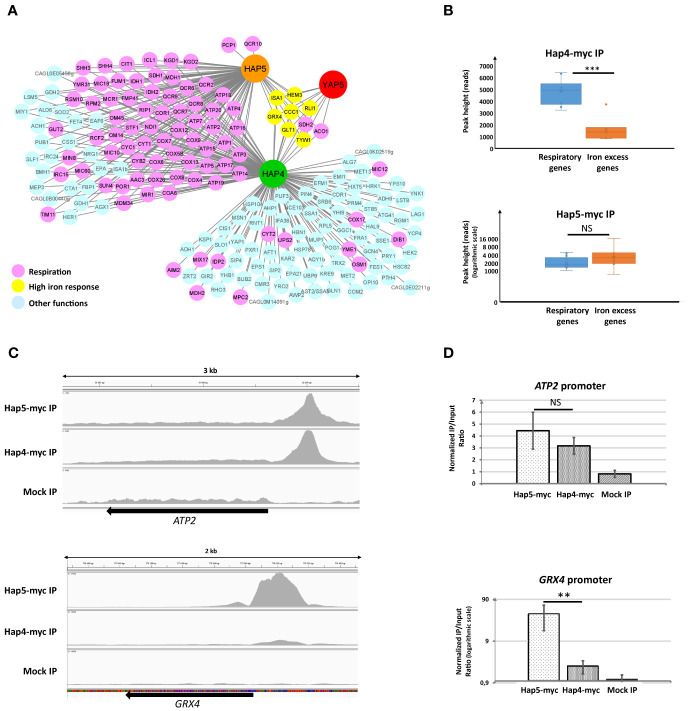
ChIP analyses of Hap4 targets. **(A)** The Hap4-Hap5-Yap5 regulatory network. An arrow indicates a potential regulatory interaction based on ChIP-seq results. The color of the targets indicates their belonging to respiratory pathways (pink), high iron response (yellow) or other pathways (light blue). The Yap5 data are from Thiébaut et al., 2017. The gene names indicated are either the official *C. glabrata* names or those of the *S. cerevisiae* orthologs, according to the CGD database (www.candidagenome.org). This representation was made using Cytoscape (Shannon et al., 2003). **(B)** Box plots of the maximal height of ChIP peaks (expressed in reads counts) corresponding to the 10 best respiratory targets (*SDH1*, *ATP2*, *FUM1*, *RPM2*, *CYT1*, *COX4*, *COX5B*, *QCR8*, *ATP5, ATP3*) (blue box plots) or the high iron tolerance genes (*GRX4*, *CCC1*, *HEM3*, *TYW1*, *ISA1*, *RLI1*, *GLT1*) (orange box plots) of Hap4 (upper panel) or Hap5 (lower panel). For the results from Hap5 IP, a logarithmic scale was used to improve the readability of the figure. The result of a t-test comparing the respiratory and iron excess genes box pots in Hap4-myc and Hap5-myc IPs is symbolized as follows: ***p < 0.01, NS: p > 0.1. **(C)** IGV snapshots of the ChIP peaks obtained for Hap5 and Hap4 IPs at the promoters of *ATP2* (upper panel) and *GRX4* (lower panel). The Y axis scales have been adjusted so that the local background (= read counts in the body of the genes) appears similar in the different lanes. The profile for the mock IP experiment (wild type, untagged strain) is also indicated. **(D)** ChIP-qPCR was performed on strains expressing either a myc-tagged Hap5 (Hap5-myc) or Hap4 (Hap4-myc). All strains were grown in glycerol media. The values on the histograms represent the IP/Input ratios of the *ATP2* (upper panel) or *GRX4* (lower panel) promoters relative to the enrichment of the *ACT1* promoter (used as an internal control). For *GRX4*, a logarithmic scale was used to improve the readability of the figure. The experiments were performed four times on biologically independent samples. Error bars hence represent the standard error of the mean. The result of a t-test comparing the Hap4-myc and Hap5-myc enrichments is symbolized as follows: **p < 0.05, NS: p > 0.1.

We identified 186 target promoters for Hap4, 99 of which were also targeted by Hap5 (**Figure 3A** and **Supplementary Table T1**). CCAAT was the most significantly enriched DNA motif in Hap4 targets, being present in 77% of the Hap4 ChIP-seq peaks. This proportion raised to 92% in the peaks shared with Hap5. Remarkably, 69% of the target promoters shared by Hap4 and Hap5 (68 among 99) were associated to respiratory genes (i.e. genes involved in respiration, TCA cycle or mitochondria functioning). This strong enrichment was in accordance with the role of the Hap4-CBC complex described in *S. cerevisiae* in the regulation of respiration and with our previous finding that Hap4 was required for the normal expression of *ATP2* in *C. glabrata* (Bolotin-Fukuhara, 2017; Thiébaut et al., 2017). More unexpectedly, we also observed significant Hap4 ChIP signals at the promoters of iron tolerance genes, i.e. at the targets of the Yap5-CBC complex (**Figure 3A** and **Supplementary Table T1**). For *GRX4*, *ISA1*, *TYW1*, *CCC1*, *HEM3, GLT1* and *RLI1*, the positions of the Hap4, Hap5 and Yap5 ChIP-peaks overlap, suggesting that Yap5 and Hap4 binds to similar locations on these promoters (**Supplementary Table T1**). Interestingly, the pattern of Hap4 binding that we observed on these promoters was quantitatively very different from the pattern that we observed at the promoters of respiratory genes, relative to Hap5. More precisely, while the ChIP-seq signal observed for Hap5 at the promoters of iron tolerance genes was equivalent to the signal measured for its 10 best respiratory gene targets, the signal measured for Hap4 at those same iron tolerance genes was clearly below the signal measured for Hap4 at its 10 best respiratory genes targets (**Figure 3B**). This observation was confirmed by visual inspection of the Hap5 and Hap4 peaks after normalization of the Hap5-ChIP and Hap4-ChIP signals on the local background (**Figure 3C**). While the Hap4 and Hap5 ChIP signals were generally of similar ranges at the promoters of respiratory genes (illustrated by the case of *ATP2* in **Figure 3C**), the Hap4 signal was comparatively much weaker than the Hap5 signal at the promoter of iron tolerance genes (illustrated by *GRX4* in **Figure 3C**). This result was validated and reproduced by ChIP-qPCR analyses of the *ATP2* and *GRX4* promoters, using ChIP samples independent from the ones used for ChIP-seq (**Figure 3D**). Again, we observed that, while the enrichments measured for Hap5 and Hap4 at the promoter of *ATP2* were of the same range, the enrichments measured for Hap5 at the promoter of *GRX4* were several orders of magnitude above those of Hap4.

**Figure 4:**
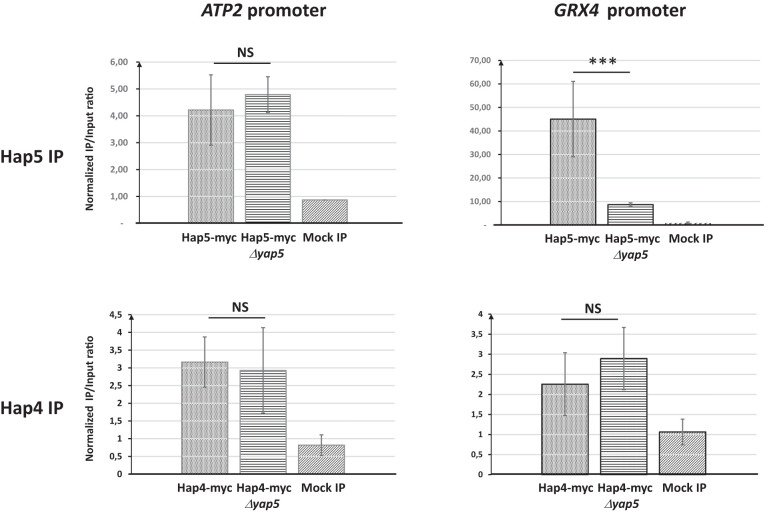
Impact of the absence of *YAP5* on the binding of Hap5 and Hap4 on the promoters of *ATP2* and *GRX4*. ChIP-qPCR was performed on strains expressing either a myc-tagged Hap5 (upper panels) or Hap4 (lower panels) in presence (Hap5-myc and Hap4-myc) or absence (Hap5-myc *yap5Δ* and Hap4-myc *yap5Δ*) of *YAP5*. All strains were grown in glycerol media. The values on the histograms represent the IP/Input ratios of the *ATP2* (left panels) or *GRX4* (right panels) promoters relative to the enrichment of the *ACT1* promoter (used as an internal control). The experiments were performed four times on biologically independent samples. Error bars hence represent the standard error of the mean. The results of a t-test comparing the enrichments obtained for the wild type *YAP5* and *Δyap5* genetic backgrounds are symbolized as follows: ***p < 0.01, NS: p > 0.1.

We next tested if the presence of Yap5 and its interaction with Hap5 at the promoters of iron tolerance genes could be responsible for this particular pattern. We conducted Hap5 and Hap4 ChIP-qPCR experiments in *C. glabrata* strains in which *YAP5* was either present or absent. We observed that, while the absence of Yap5 had no impact on the Hap4 ChIP-qPCR signal at *GRX4* and *ATP2* promoters or on the Hap5 ChIP Q-PCR signal at *ATP2* promoter, it dramatically reduced the enrichment of Hap5 at the promoter of *GRX4* (**Figure 4**). Actually, in the absence of Yap5, the enrichments of Hap5 and Hap4 at the *GRX4* promoter were of the same range, mimicking the situation observed for respiratory genes (**Figure 4**). Prior to these experiments, we had controlled that the deletion of *YAP5* had no impact on the expression levels of the Hap5-myc and the Hap4-myc proteins (**Supplementary File S9**).

As conclusion to this section, Hap4 is found at the promoter of both respiratory genes and iron tolerance genes. However, the Hap4/Hap5 ChIP signal ratio at these two gene categories is very different. On respiratory genes, this ratio is close to one, which is consistent with the stoichiometric Hap4-CBC interaction described in *S. cerevisiae* for the regulation of those genes (McNabb and Pinto, 2005). In contrast, on iron tolerance genes, this ratio is very low, suggesting that Hap4 is not the main partner of CBC at these promoters. This situation is mostly due to Yap5, since in its absence, the ChIP enrichment of Hap5 drop down to reach similar values than Hap4 at these promoters. In other terms, in the absence of Yap5, the pattern of Hap4 and Hap5 binding to iron tolerance genes observed by ChIP becomes very similar to the one observed at the promoters of respiratory genes.

### GRX4 Expression Is Regulated by Hap4 Only When Yap5 Is Absent

The Hap4 ChIP-seq results challenged our previously published model that Hap4 does not contribute to the high iron response (Thiébaut et al., 2017). To get a genome-wide view of the role of Hap4 in the regulation of gene expression in *C. glabrata*, we performed transcriptome comparisons of a wild type strain and of a strain deleted for *HAP4*, grown either in glycerol as sole carbon source or in glucose with iron excess. We compared the results with transcriptome profiles obtained for a *Δhap5* strain grown in the same conditions and with a *Δyap5* strain grown in iron excess conditions.

**Figure 5:**
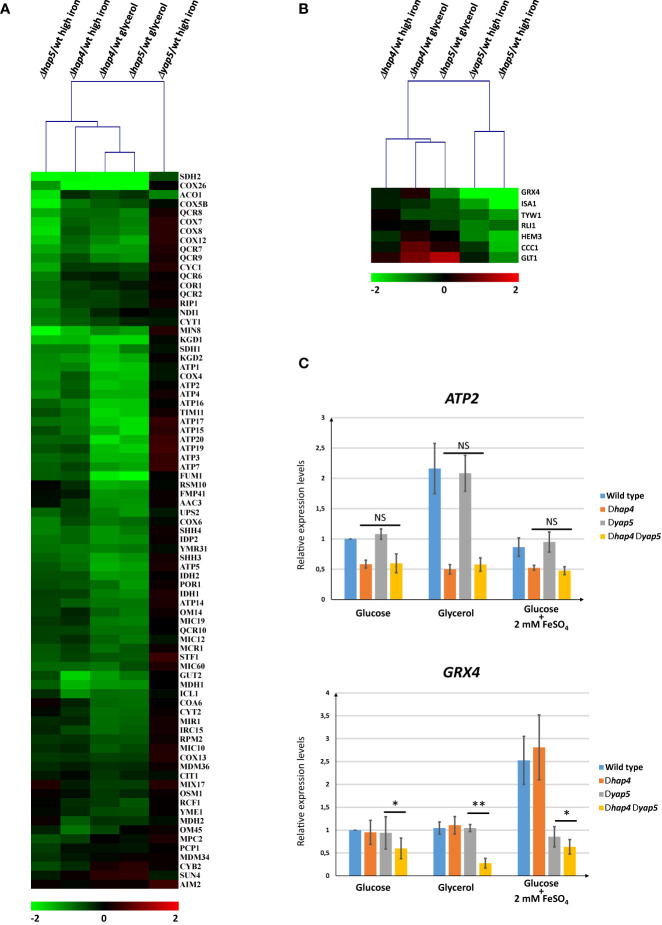
Expression analyses of Hap4 gene targets in different growth conditions and genetic contexts. **(A, B)** Transcriptome comparisons of Hap4, Hap5 and Yap5 impact on gene expression. The *Δhap4*, *Δhap5* and *Δyap5* strains were grown in different conditions (glycerol media or glucose media with iron excess (2 mM FeSO_4_) and their transcriptomes were compared to wild type cells grown in the same conditions using microarrays. The values represented on the eisengrams are log2 of mutant/wild type expression ratios. The color scale is indicated at the bottom of each eisengram. Hierarchical clustering using correlation distance was used to cluster the samples based on the expression profiles of either **(A)** the respiratory genes (as defined in ) or **(B)** the iron tolerance genes (as defined in ). The Yap5 and Hap5 high iron data are from Thiébaut et al., 2017. **(C)** Analyses of the impact of a double deletion of *HAP4* and *YAP5* on the expression of *ATP2* (upper histogram) and *GRX4* (lower histogram). The relative expression of *ATP2* and *GRX4* was measured by RT-qPCR in wild type, *Δhap4Δyap5* and *Δhap4Δyap5* strains grown in glucose, glycerol or glucose + iron excess (2 mM FeSO_4_). The values represent the expression levels of the *ATP2* or *GRX4* genes relative to *ACT1* (used as an internal control) and to the wild type grown in glucose, which was arbitrarily set to 1. The experiments were performed six times on biologically independent samples, except for glycerol which was based on three replicates only. Error bars represent the pearson standard deviation. The results of a t-test comparing the expression levels of *ATP2* between the *Δhap4* and the *Δyap5Δhap4* strains (upper panel) or the expression levels of *GRX4* between the *Δyap5* and the *Δyap5Δhap4* strains (lower panel) are symbolized as follows: *p < 0.1, **p < 0.05, NS: p > 0.1.

The results of these global analyses confirmed our previous model (**Figures 5A, B** and **Supplementary Table T2**). We observed that, like Hap5, Hap4 was required for the normal expression of respiratory genes in the three growth conditions that we tested. As described previously (Thiébaut et al., 2017), the deletion of *YAP5* had no impact on the expression of those genes, with the sole exceptions of *SDH2* and *ACO1*, which belong to the respiratory genes and to the iron tolerance genes categories (**Figure 5A**). Reciprocally, the deletion of *HAP4* did not impact the induction of the iron tolerance genes in high iron conditions, while the deletion of *HAP5* or *YAP5* had a dramatic effect on the high iron response (**Figure 5B**). These observations were supported by hierarchical clustering of the experimental conditions based on gene expression. When only the expression patterns of the respiratory genes were taken into account, the *Δhap4* samples clustered with the *Δhap5* samples and the *Δyap5* sample was clearly an outlier (**Figure 5A**). When only the expression patterns of the iron tolerance genes were used for samples clustering, the *Δhap5* high iron and *Δyap5* high iron conditions clustered together, while the *Δhap4* samples and the *Δhap5* glycerol conditions were in a second group (**Figure 5B**).

Although these results confirmed that Hap4 was not required for proper high iron response, the ChIP-qPCR results reported in the previous paragraph suggested that the situation could be different in the absence of Yap5. Indeed, we had showed that, when *YAP5* is deleted, the Hap4/Hap5 ChIP pattern at the *GRX4* promoter tends to be similar to the one observed at the *ATP2* promoter (**Figure 4**). In other terms, we wondered if the absence of Yap5 could turn the *GRX4* expression regulation from a “iron tolerance gene-like” mode into a “respiratory gene-like” mode.

To address this question, we constructed a double knock-out *C. glabrata* strain in which both *YAP5* and *HAP4* were deleted. We then analyzed by RT-qPCR the expression of *GRX4* and *ATP2* in the wild type, simple mutants or double mutant strains, grown in the presence of either optimal or toxic iron concentrations, or with glycerol as the sole carbon source (**Figure 5C**). As expected from our previous work and from the transcriptome analyses presented in **Figures 5A** and **B**, the simple deletion of *HAP4* constitutively decreased the expression of *ATP2* and abolished its induction by glycerol, but it had no impact on the expression levels of *GRX4*. Reciprocally the deletion of *YAP5* severely impaired the high iron induction of *GRX4* but had no effect on the expression of *ATP2*.

The expression levels of *ATP2* in the double *Δhap4 Δyap5* mutant were not different from the simple *Δhap4* mutant, confirming that Yap5 is not involved in the regulation of this gene (**Figure 5C**). In contrast, the deletion of *HAP4* in a *Δyap5* context decreased the expression of *GRX4* as compared to the *Δyap5* simple mutant. This effect was iron independent, since it was similarly observed in normal and high iron growth conditions. Interestingly enough, this effect seemed to be more important in glycerol grown cells, which is the condition at which Hap4 is supposed to have higher activity and level of expression.

To check if this effect was specific to *GRX4* or could be generalized to other Yap5 targets, we analyzed in the same strains the expression of *CCC1* and *ISA1*. For these two genes, we did not observe the additive effect of the *HAP4* deletion in a *YAP5* deleted background (**Supplementary File S10**). For these two genes, the absence of Yap5 is not enough to allow regulation by Hap4, in contrast to what was observed for *GRX4*.

As a conclusion to this section, the presence of Yap5 seems to prevent Hap4 to act on the expression of *GRX4*. In the absence of *YAP5*, Hap4 exerts a constitutive, positive, effect on *GRX4* expression, which is reminiscent of its activity on respiratory genes. Yet, *GRX4* does not behave exactly like a respiratory gene in a *Δyap5* context, as shown by the fact that *GRX4* expression level are not increased by glycerol in the *Δyap5* simple mutant. Moreover, this conclusion cannot be generalized to all Yap5 targets, as the levels of expression of *CCC1* and *ISA1* do not decrease when *HAP4* is deleted in a *Δyap5* background.

## Discussion

Gene duplication is a major source of functional diversification (Innan and Kondrashov, 2010; Voordeckers et al., 2015). However, the co-existence of two paralogues with similar biochemical properties but diverging functions can lead to potentially detrimental competition between the duplicates. This phenomenon has been named “paralogue interference” and is particularly relevant in the case of transcriptional regulators, which work very often as cooperative complexes (Baker et al., 2013). In previously described cases, this paralogue interference constraint was overcome by mutations in one or both paralogues, which prevented or biased the competition between them to allow sub-functionalization without losing important functions under strong selection pressure (Bridgham et al., 2008; Baker et al., 2013; Pougach et al., 2014). In many cases, this led to a complexification of the derived regulatory networks and, potentially, to the appearance of new regulatory modes and new functions (Voordeckers et al., 2015).

**Figure 6:**
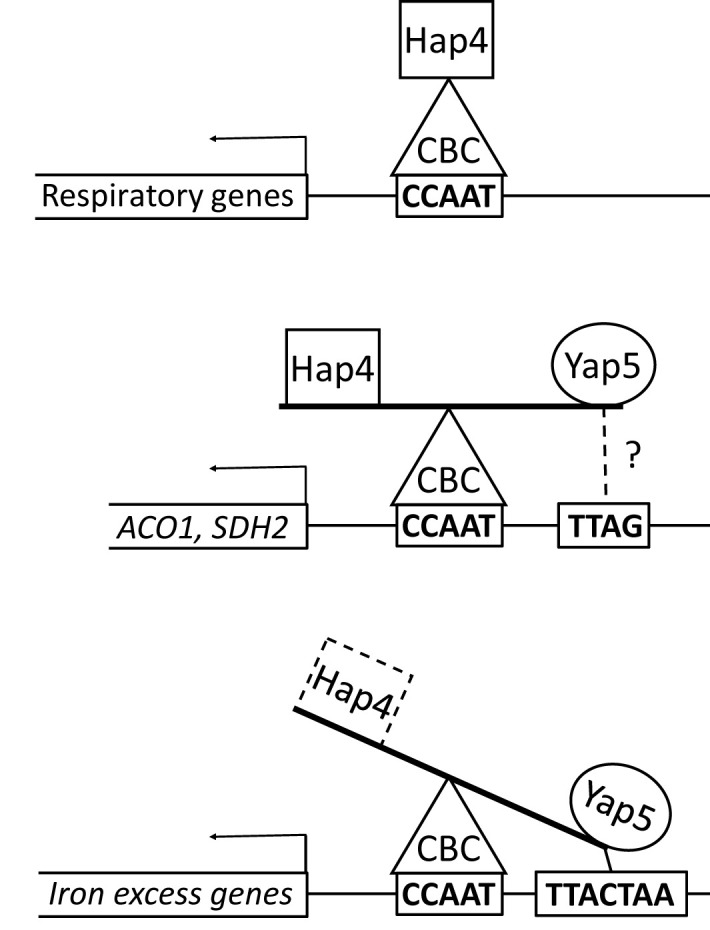
The Hap4/Yap5 balance at the promoters of CBC targets. Hap4 is found at the promoters of all CBC targets, probably because of the strong affinity of its perfect Hap4L motif for Hap5. However, at the promoter of iron excess genes (lower panel), the preferred regulatory partner of CBC is Yap5, probably because of the presence of a YRE close to the CCAAT box, which strengthens the CBC-Yap5-DNA interaction. For *ACO1* and *SDH2* (middle panel), the CCAAT surroundings are less favorable to Yap5 and then the competition with Hap4 is more balanced. Additionally, for *ACO1*, a second Hap4 ChIP peak is detected on a CCAAT rich region upstream of the Yap5 peak (not shown on this figure).

In the present work, we addressed this question by studying the interplay between Yap5 and Hap4, two regulatory subunits of the CBC in *C. glabrata*. Yap5 and Hap4 are not considered as paralogues *per se*, because their sequence similarity is very low. However, they both have a Hap4L domain which confers them the ability to interact with the CBC, making each other potential competitors for the regulation of CBC targets. Still, it was shown previously that the Yap5 and Hap4 pathways seemed to work independently (Thiébaut et al., 2017). In other terms, mechanisms evolved to avoid detrimental interference between these two regulatory networks. Based on a work on HapX in *A. fumigatus*, which showed that the C-terminal part of the Hap4L domain is involved in low specificity DNA binding and target selection of the HapX-CBC complex, it was proposed that Hap4 could similarly distinguish between iron tolerance promoters and respiratory genes in *C. glabrata* (Furukawa et al., 2020). Actually, our observations do not exclude a potential contribution of Hap4 to DNA target recognition, but they do not go into that direction either. Especially, our ChIP-seq experiments showed that Hap4 can be found at the promoters of both respiratory and iron tolerance genes, suggesting that it is not able to clearly discriminate between the two types of genes. Our results rather suggest that Yap5 is the main actor of the selectivity that is observed in this system. Yap5 seems to be dependent, not only on its interaction with the CBC bound to the CCAAT box, but also on the presence of a YRE located at 10 to 14 bp of the CCAAT box. This functioning of Yap5 is very probably the same in most *Saccharomycetaceae* species, as the CCAAT-YRE bipartite motifs in *GRX4, CCC1, ISA1* and *TYW1* promoters were remarkably conserved in this clade. This strict DNA binding specificity physically restricts Yap5 regulation to a handful of genes containing a bipartite CCAAT-YRE DNA motif and probably explains why Yap5 is not able to efficiently compete with Hap4 for the regulation of respiratory genes, which do not contain this bipartite motif. Conversely, this strict DNA binding specificity seems to provide Yap5 with an advantage in its competition with Hap4 for the regulation of iron tolerance genes. Hence, the presence of Yap5 seems to strengthen the binding of Hap5 to the promoter of *GRX4*. Moreover, according to our results, this is the presence of Yap5 which prevents Hap4 to act on the expression of *GRX4*, although this effect was not observed for other Yap5 targets, which suggests that other parameters besides the presence of Yap5 interfere with a potential Hap4 regulation of those genes. The only exceptions to this model are *SDH2* and *ACO1*, which only exhibit a half-YRE motif close to the CCAAT box (TTAGT and TTAG, respectively) in their promoters but yet are bound and induced by Yap5 in response to iron excess. Interestingly, *SDH2* and *ACO1* have a hybrid regulation, being both respiratory genes controlled by Hap4 and part of the high iron response induced by Yap5. Hence, an equilibrium seems to have been selected for those two genes in the competition between Yap5 and Hap4, which would have been compromised by the presence of a perfect CCAAT-YRE in their promoters. In summary, the Yap5/Hap4 competition appears to be a quantitative and dynamic balance which is controlled by their respective capacity to interact with CBC on the one hand, and by the more or less strict specificity of Yap5, and possibly Hap4, for the DNA sequences surrounding the CBC CCAAT binding sites on the other hand (**Figure 6**). Quantitative biochemical analyses of the Yap5-CBC-DNA and of the Hap4-CBC-DNA interactions using techniques such as surface plasmon resonance (Hortschansky et al., 2007; Hortschansky et al., 2015; Furukawa et al., 2020) will be required to better understand the molecular basis of this balance.

As mentioned in the introduction, although their similarity over their whole sequences is relatively weak, Yap5 and HapX have the same functional domains, i.e. a HAP4L domain, a bZip motif and CRDs (Merhej et al., 2015; Rietzschel et al., 2015; Thiébaut et al., 2017). Hence, although their roles in the regulation of iron homeostasis have significantly diverged, they very likely derive from a common ancestor and are, therefore, probably orthologues. Still, their DNA binding properties look significantly different. HapX has been shown to contribute to the target selectivity of the HapX-CBC complex with a relatively low specificity. More precisely, HapX-CBC recognizes a tripartite motif composed of a CCAAT box and of two motifs, RWT and TKAN, positioned at 12 bp and 11 to 23 bp from the CCAAT sequence, respectively (Furukawa et al., 2020). Some versions of the TKAN motif resemble a half YRE site, but clearly HapX does not require the presence of a canonical YRE to bind DNA and regulate gene expression (Hortschansky et al., 2015; Furukawa et al., 2020). So, since their bZip sequences are very similar, why are Yap5 and HapX DNA binding properties different? Based on the available data, at least two complementary hypotheses can be proposed. First, the last four amino acids at the C-terminus of Yap5 Hap4L domain have diverged from the canonical sequence found in HapX (Merhej et al., 2015). This truncated Hap4L may weaken the interaction of Yap5 with CBC and/or with the DNA sequences surrounding the CCAAT (Furukawa et al., 2020). Hence, the specific interaction with a YRE close to the CCAAT may act as a compensatory mechanism to increase the CBC-Yap5-DNA binding energy. In support of this hypothesis, we observed in a previously published study that, while the deletion of *HAP5* or of the whole Hap4L in *YAP5* completely abolished Yap5 binding to *GRX4*, single mutations in important positions of the Hap4L domain in Yap5 severely diminished its interaction with *GRX4*, but a significant ChIP enrichment was still detectable [**Figure 4B** in Thiébaut et al. (2017)]. This result suggests that, in the presence of a CCAAT-YRE DNA motif, non-optimal Yap5-CBC interactions can be sufficient to ensure a minimal binding efficiency of Yap5 to its targets. Second, HapX is, to the best of our knowledge, the only Hap4L protein encoded by the *A. fumigatus* genome, while Yap5 had to cope with the potential competition of Hap4 for CBC binding and gene target regulation. Hence, as discussed above, the accumulation of mutations in Yap5 Hap4L domain on one hand and the presence of a bipartite CCAAT-YRE motif in the promoters of the Yap5-CBC targets on the other hand, may have been selected to resolve this potential conflict between the two CBC regulatory subunit, by providing an advantage to Yap5 over Hap4 at the promoters of the iron tolerance genes, while disadvantaging Yap5 at the promoters of respiratory genes. In this matter, it would be very interesting to study the interplay between Hap4L containing proteins in non-*Saccharomycetaceae* yeast species such as *C. albicans*. In this species, there are two Hap4 homologues (Hap41 and Hap42) and one Hap4L-bZip protein (Hap43) which is clearly more similar to HapX than to Yap5, both for its sequence and for its functioning (Hsu et al., 2011; Singh et al., 2011; Merhej et al., 2015). So, other solutions must have been selected to avoid competition between these three Hap4L proteins. Unfortunately, the roles of Hap41 and Hap42 are largely unknown and Hap43 DNA interaction properties have not been investigated in detail. Hence, we have little information to understand the mechanisms at play in this case, besides the fact that Hap43 can act as a repressor of the expression of *HAP41* (Chen et al., 2011).

The hypothesis of a co-evolution of the Hap4 and Yap5 regulons shed a new light on the evolution of the respiratory and iron homeostasis regulatory networks in yeast. Interestingly enough, the yeast species which have a Hap4L-bZip protein of the Yap5 type (i.e. *Saccharomycetaceae*), are also the ones strongly enriched for crabtree positive species (Dashko et al., 2014; Hagman and Piskur, 2015). Moreover, the *Saccharomycetaceae* species in which the CCAAT-YRE motif was less conserved, i.e. *Kluyveromyces sp* and *Eremothecium sp* (**Figure 2A**), are the ones which are less prone to use crabtree metabolism according to Hagman et al. (Hagman et al., 2013). The crabtree effect is defined as the capacity to favor fermentation over respiration in the presence of sugars, even in aerobic growth conditions. The induction of the respiratory genes by Hap4 is an important aspect of the crabtree metabolism (Perez-Samper et al., 2018). Hence, by allowing the divergence of the regulation of the respiratory genes and the iron tolerance genes, the resolution of the potential interference between Hap4 and Yap5 possibly played a key role in the fixation of this new metabolic feature in *Saccharomycetaceae*. Reciprocally, it is tempting to speculate that the positive selection of this new regulation of respiration has been a strong driving evolutionary force for the specialization of Yap5 in the regulation of the high iron response in these species. A collateral consequence of this process was that Yap5 lost its role in the repression of iron consuming genes upon iron starvation, which is the common feature of all other HapX homologues (Devaux and Thiébaut, 2019). However, this regulation, which is crucial for the adaptation to iron scarce conditions, is ensured at the post-transcriptional level by the Cth2 RNA binding protein in extent *Saccharomycetaceae* species, which compensate for the absence of a “HapX-like” regulation of those genes (Puig et al., 2005; Gerwien et al., 2016).

In conclusion, we showed that Hap4 has the potential to interfere with the regulation of Yap5, since Hap4 is found at the promoters of both respiratory genes and iron tolerance genes. This potential interference between the two regulatory pathways is minimized by the strict DNA binding specificity of Yap5 for CCAAT-YRE motifs, which, on one hand prevent Yap5 to bind to most respiratory genes and, on the other hand, strengthen the Yap5-CBC-DNA interaction at the promoters of iron tolerance genes. Consequently, the Hap4 and Yap5 pathways seem to work independently in *C. glabrata*. Hence, the resolution of the Hap4/Yap5 interference led to the diversification of the regulatory network in *C. glabrata*, and probably in most *Saccharomycetaceae* species, by allowing the acquisition of a Hap4-dependent regulation of respiration, while considerably restricting the set of targets of Yap5 as compared to its HapX homologues. We propose that the positive selection of a new metabolic capacity, i.e. the crabtree effect in the *Saccharomycetaceae*, has shaped the evolution of the iron homeostasis regulatory network in budding yeasts.

## Data Availability Statement

The datasets presented in this study can be found in online repositories. The names of the repository/repositories and accession number(s) can be found below: https://www.ebi.ac.uk/arrayexpress/, E-MTAB-https://www.ebi.ac.uk/arrayexpress/, E-MTAB-10675, E-MTAB-10544.

## Author Contributions

TD performed the ChIP-Q-PCR and RT-Q-PCR analyses, constructed the strains and performed the directed mutagenesis of the *GRX4* promoter. MB and AT performed the ChIP-seq experiments. AT performed the ChIP-seq raw data treatment and FD did the peak calling step. AT and FD performed the *GRX4* multispecies sequence analyses. FD performed the microarray experiments. AT did the RT-Q-PCR analyses on the *LacZ* reporter. JM constructed the pSG plasmid and wore a PSG tee-shirt for most of his post-doc. FD interpreted the results, submitted the sequencing and microarray data to public repositories, made the figures and wrote the manuscript. All authors contributed to the article and approved the submitted version.

## Funding

This work was funded by the Agence Nationale pour la Recherche (CANDIHUB project, grant number ANR-14-CE14-0018-02).

## Conflict of Interest

The authors declare that the research was conducted in the absence of any commercial or financial relationships that could be construed as a potential conflict of interest.

## Publisher’s Note

All claims expressed in this article are solely those of the authors and do not necessarily represent those of their affiliated organizations, or those of the publisher, the editors and the reviewers. Any product that may be evaluated in this article, or claim that may be made by its manufacturer, is not guaranteed or endorsed by the publisher.
