# Supplementary material for: Yap5 Competes With Hap4 for the Regulation of Iron Homeostasis Genes in the Human Pathogen Candida glabrata
Source: Front Cell Infect Microbiol. 2021 Nov 26;11:731988. doi: 10.3389/fcimb.2021.731988 (PMC8662346; doi:10.3389/fcimb.2021.731988)
Supplement: Supplementary file 1 [file DataSheet_1.pdf]

Supplementary file S1: Primers used in this study.

NAME	SEQUENCES (5'=>3')	Use
301-PqCgAP6-F	GGGGAATTGTGGGTTCTGTA	Verification of *yap5Δ*
300-CgAP6ter-Rev	CCACACTAGTAATGTGGAGAT	Verification of *yap5Δ*
296-CgAP5pro-F1	AATGCCCTCACTTGGGTGTAAAATAGTATATATATGTGTGTAGTGCTTAGTAGCTGTTATGTAGTGTGAGGACGATGTGTCGGATCCCCGGGTTAATTAA	Deletion cassette for YAP5
2032-CgAP6ter-R1-Corrigé	ATTAAGAACAATGAATCTATCAGGTAAAAGTTTGTATTTATAAGATATTATGAATATATTAAGCTATTCTACAGCGATAAGAATTCGAGCTCGTTTAAAC	Deletion cassette for YAP5
880-HAP4TermVerifDelRev	GGTGGACAGTACTGGCATTA	Verification of Hap4 myc tagging
852HAP4EndMycFor	GTTTAATGACCCTTCAAATACTGCTAGACAACAAGATGACCTAATTGTTGACACTGATGGTGACATGTTTGCATTTATTCGGATCCCCGGGTTAATTAA	Myc Tagging of Hap4
853HAP4TermMycRev	AATAGGCTAGGCTTCTACTCTAGGTGAAGCACGAGCCATTTTTTTATCCTTTGATCTCTTCCAATATAAAATAAAAACAGAATTCGAGCTCGTTTAAAC	Myc Tagging of Hap4
908-PromCgGRX4-SacII-SmaI-PrF	GAATCCGCGGCCCGGGTACCAAGGGATACACTTGCAAATGC	cloning of *GRX4* promoter in pSG
909-PromCgGRX4-NotI-PrR	GATAGCGGCCGCTGCCCCCCACCATCACTGCCAGACTCTCAACTG	cloning of *GRX4* promoter in pSG
910-PromCgGRX4MutYRE-PrF	CGATGGTGGCGATTCAGGATGCACTCGACG	mutagenesis of the YRE in *GRX4*
911- PromCgGRX4MutYRE-PrR	CGTCGAGTGCATCCTGAATCGCCACCATCG	mutagenesis of the YRE in *GRX4*
912-PromCgGRX4MutCCAAT-PrF	GCACTCGACGGTGATTTTCTGAGACCGAGAC	mutagenesis of the CCAAT in *GRX4*
913- PromCgGRX4MutCCAAT-PrR	GTCTCGGTCTCAGAAAATCACCGTCGAGTGC	mutagenesis of the CCAAT in *GRX4*
854HAP4ORFSeqVerifFor	GATGAACAAGACGCGCACTCC	Sequencing of Hap4 myc tagging
329-HIS5-For	GCAAACCAAAAGGGAGAACA	Verification of HIS marker insertion
330-HIS5-Rev	GGACAATTCCCCAACCTTTT	Verification of HIS marker insertion
464-CAGL0G08151gPr-F	AACCAGCAAACCAGGAACAC	ChIP-QPCR GRX4
465-CAGL0G08151gPr-R	TGCATTACTAATCGCCACCA	ChIP-QPCR GRX4
2020-CgATP2promF	GAGGCAGTCCACATCGCAG	ChIP-QPCR ATP2
2021-CgATP2promR	GGATTCGAGGATTTCCCAAC	ChIP-QPCR ATP2
466-Pq-CAGL0G08151g-F	TCAGATTCGGGTTCTTCGAC	RT-QPCR GRX4
467-Pq-CAGL0G08151g-R	TAGGCCACCTTGGAACTCAC	RT-QPCR GRX4
885-CgATP2-Q-for	CCATTCGCTGTCGCTGAAG	RT-QPCR ATP2
886-CgATP2-Q-rev	GCAACGACATCTTCAATACC	RT-QPCR ATP2
CgACT1-Pr-F	AAAGCGATTGCAAACTTCC	control for ChIP-QPCR
CgACT1-Pr-R	CAGAACCCGTCACCAAAA	control for ChIP-QPCR
Q-CgACT1-For	GCTCCAGAAGCTTTGTTCCACCCAT	control for RT-QPCR
Q-CgACT1-Rev	CATCACACTTCATGATGGAGTTGTA	control for RT-QPCR
78- Q_laczfor	CGTCACGAGCATCATCCTCTGC	RT-QPCR LacZ
79- Q_laczrev	GTCGCACAGCGTGTACCACAGC	RT-QPCR LacZ

Supplementary file S2: Strains used in this study.

Strain name	Parental strain	Genotype	Origin
**HTL**	ATCC 2001	his3Δ/trp1Δ/leu2Δ	Schwartzmuller et al., 2014
**hap4Δ**	HTL	CAGL0K08624g::NAT/his3Δ/trp1Δ/leu2Δ	Schwartzmuller et al., 2014
**hap5Δ**	HTL	CAGL0K09900g::NAT/his3Δ/trp1Δ/leu2Δ	Schwartzmuller et al., 2014
**yap5Δ**	HTL	CAGL0K08756g::HIS5/his3Δ/trp1Δ/leu2Δ	this work
**hap4Δyap5Δ**	hap4Δ	CAGL0K08624g::NAT/CAGL0K08756g::HIS5/his3Δ/trp1Δ/leu2Δ	this work
**Hap5-myc**	HTL	CAGL0K09900g-13Myc-HIS5/his3Δ/trp1Δ/leu2Δ	Thiebaut et al., 2017
**Hap4-myc**	HTL	CAGL0K08624g-13Myc-HIS5/his3Δ/trp1Δ/leu2Δ	This work
**Hap4-myc, yap5Δ**	HTL	CAGL0K08624g-13Myc-HIS5/CAGL0K08756g::TRP1/his3Δ/trp1Δ/leu2	This work
**Hap5-myc, yap5Δ**	hap5Δ	CAGL0K09900g-13Myc-HIS5/CAGL0K08756g::TRP1/his3Δ/trp1Δ/leu2	This work
**HTL, prom-GRX4mutCCAAT-LacZ**	HTL	his3Δ/trp1Δ/leu2Δ, pSG-PromGRX4mutCCAAT-LacZ-HIS3	This work
**HTL, prom-GRX4mutYRE-LacZ**	HTL	his3Δ/trp1Δ/leu2Δ, pSG-PromGRX4mutYRE-LacZ-HIS3	This work
**HTL, prom-GRX4LacZ**	HTL	his3Δ/trp1Δ/leu2Δ, pSG-PromGRX4-LacZ-HIS3	This work

(A)

Species	Sequence (←)	Group
S. cerevisiae	ACCGAG**CCAAT**GAGAAGAGGGTATG**TTAGTAA**TATTAGTG	Yap5-like (*Saccharomycetaceae*)
S. mikatae	ACTGAG**CCAAT**GAGAAATGAGTACC**TTAGTAA**TATTCGTG	
S. kudriazevii	GCTGAA**CCAAT**GAGAGGCCGGCGCG**TTAGTAA**TAGTTGCG	
S. bayanus	GCTGGA**CCAAT**GAGAGGGGAGCGCG**TTAGTAA**TCGGCGCA	
C. glabrata	CAACGC**CGAAT**GGCAGCCGAGCAGC**TTACTAA**GAGCTCGT	
N. delphensis	TGACAG**CCAAT**AGTTGCAGTGTTTA**TTAGTAA**GAGAGATC	
C. nivariensis	GTTCAG**CCAAT**GAAATCGAAGAATG**TTAGTAA**GTCAAATC	
C. bracarensis	ATTGAG**CCAAT**GAAATAACATTCTG**TTAGTAA**TGCAGGTA	
C. castelli	TTTCAC**CCAAT**CAAAACAAAGGAAA**TTACTAA**TCTTCAGT	
N. bacillisporus	ATTCGA**CCAAT**CAGATCCGAGAACAGTGGTAA**TTAC**AATG	
K. naganishii	TTGAGG**CCAAT**GAAGTCAGAGTGTA**TTAGTAA**TGGGAAAT	
K. africana	AAGCAG**CCAAT**AGATTCAGTAGTCGTCCGTGTACA**TTAGTAA**	
T. blattae	TTTTAG**CCAAT**CGGAATTGTCAAA**TTAGTAA**TATTAAGAC	
T. Phafii	ATTATTAGTTGATGGATTAGGAGGA**TTAGTAA**TTTCTGCG	
N. castellii	GTCCGG**CCAAT**CAGAAGCGAGATTG**TTACTAA**TAACTTAC	
Z. rouxii	GGTTAG**CCAAT**TAAAAGTGCCTTTA**TTACTAA**GGCATGTG	
Z. bailii	GACTGA**CCAAT**GAGCATCAACTTCA**TTACTAA**GACCTGTT	
T. Microellipsoides	CATTGA**CCAAT**CAAAACCGAGAACG**TTACTAA**TAGAACAT	
L. fermentati	TTTTGA**CCAAT**GAGAGAATGTTCTA**TTAGTAA**AAACAAGA	
L. thermotolerans	AACTGG**CCAAT**CAGGAAAAGTTTCA**TTACTAA**GAGAGCGG	
L. quebecensis	GACTGG**CCAAT**CAGGAAAACTTTCA**TTACTAA**GGGAGCGA	
L. mirantina	TATTGG**CCAAT**CAGGATTGGCTTCA**TTAGTAA**TGGATAAT	
L. dasiensis	GTACGA**CCAAT**AAGAATCGTTTTCA**TTAGT**C**A**CCCAACGA	
L. meyersii	GTACGG**CCAAT**CAGAAGCCTGCTCA**TTAGTAA**AACAACAA	
L. nothofagi	AAACTA**CCAAT**GGCTGGTACAAATCG**T**A**AGT**G**A**TTTATTT	
L. kluyveri	CGACGA**C**A**AAT**GAGAGAATGCATAA**TTACTAA**GCTTGTAA	
L. lanzarotensis	TACGAA**CCAAT**CACAAGCGTTTTCA**TTAGTAA**AAGGCTCA	
K. lactis	CTTGTT**CCAAT**ATTGTTTCTCTAAAAACTAAATACAGAAA	
K. marxianus	GCAACG**CCAAT**GCTAATTACCAGAGTACGGTAGGTAACAG	
E. cymbalariae	CAAAAA**CCAAT**AAATTATTCAAAGGGGGCGGTGGGTGGCC	
E. gossypii	ACAGCGGC**TTACTAA**CGCCGGGAAATGTG**ATTGG**CCCGAC	
C. fabianii	GTATTG**CCAAT**ACGACAAATTCTTTAGCAATAACATGCGT	HapX-like
M. farinosa	AGGTGA**CCAAT**GAAAGTTACGAAATTTATAAGCAATTGCA	
D. hansenii	GGGATT**CCAAT**CCCCATTAGGTACATCCTTACACCAAGAG	
C. parapsilosis	ACGGAT**CGAAT**AAGAAAGAAAAAAGTGAAGTGAATTGGGT	
C. albicans	TTTCCA**CCAAT**AATTGCAGTTCACCAATGTATTATTACGT	
A. adeninivorans	TTCTGA**CCAAT**AATATTGCGCCTAATTCCTTGATCCCATG	
Y. lipolytica	TGGAATGAT**TTAGTAA**TGCGCGGCCTGTG**ATTGG**CCCGCC	

(B)

CCC1

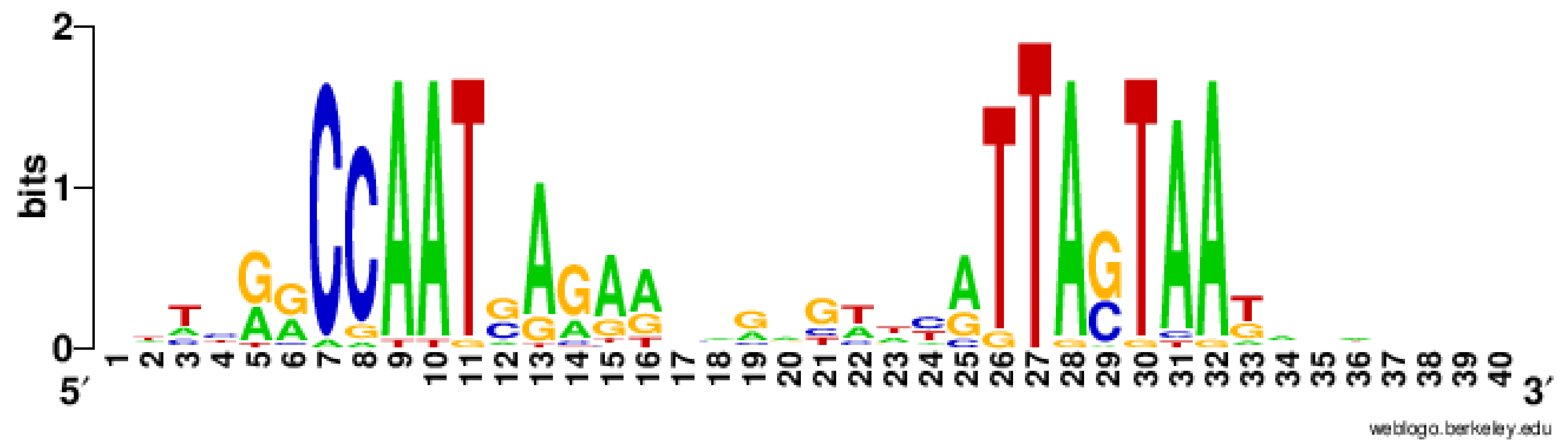

Supplementary file S3: Conservation of the CCAAT-YRE bipartite motifs in *CCC1* orthologues from 39 Hemiascomycetes yeast species. (A): Alignment of the CCAAT-YRE bipartite motifs. The sequences were arbitrarily aligned based on the CCAAT box, except for *E. gossypii* and *T. phafii*. All sequences are oriented 5’-3’ from left to right. The arrow on top indicate the sense of transcription of *CCC1*, i.e. the arrow oriented to the left indicates that the CCAAT-YRE motif is on the minus strand. (B): Seqlogo of the CCAAT-YRE alignments in the sequences from the *Saccharomycetaceae* species (“Yap5-like” species) presented in panel (A), excluding *N. bacillisporus, K. lactis, K. marxianus, E. cymbalariae* and *E. gossypii*.

(A)

Species	Sequence (← sense of transcription of *ISA1*)	Group
S. cerevisiae	ATCTGA**CCAAT**GAAATCTGAGGGTA**TGACTAA**TAAGCCCC	Yap5-like *(Saccharomycetaceae)*
S. mikatae	TTCTGA**CCAAT**AAAAGCTGAGGATG**TGACTAA**TGGCCTCG	
S. kudriazevii	TTTTGA**CCAAT**CAGAGGCAAATGTG**TGACTAA**TGAACTCC	
S. bayanus	CTCCAG**CCAAT**CAGAGCCGAGGATA**TGACTAA**TTGGCTCG	
C. glabrata	ACTGGG**CCAAT**GAGGTACGAGTCTG**TGAGTAA**GAAAACGA	
N. delphensis	AGCGAG**CCAAT**GAAAATCGAGTAAA**TTAC**C**TA**ATATTAAT	
C. nivariensis	AGCGAG**CCAAT**AAGAATCGAGTGTA**TTAGTTA**TTTATGCA	
C. bracarensis	TGCGAG**CCAAT**GAAAATTGAGTGTA**TTAGTTA**TAATATAG	
C. castelli	GACTGT**CCAAT**CGCAAACCCCAAACTCGCGACCGCGAGTA	
N. bacillisporus	GTTGGA**CCAAT**GACCGTCGAGCCTG**TTACATA**CACAGCGG	
K. naganishii	CGACAG**CCAAT**GAAAGAGAAGACTG**TTACAAA**CGCACTTG	
T. blattae	CGGGAG**CCAAT**GAAATTGAAGAACG**TTACAAA**TAACCTTG	
N. castellii	ACTCGA**CCAAT**CAGCGGCGAGTTTC**TTAGTAA**ATTTGTCG	
Z. rouxii	ACATCA**CCAAT**ATTCATGATCCTCAACATGAAAACTACCG	
Z. bailii	ATGCCC**CCAAT**TCAAATTGCGGACCGAGAGTCGGCATTGG	
T. Microellipsoides	AAATGA**CCAAT**CATAAGCCAGCGTA**TTACAAA**CACTTATT	
L. fermentati	TGACAG**CCAAT**CATATCAAAATGTA**TTAGTCA**CAATTACA	
L. thermotolerans	ATTTGG**CCAAT**TAGAAGGCCTTTCT**TTACTAA**GGGCGCCT	
L. quebecensis	ACCTGA**CCAAT**CAGGGTCCCTTTCT**TTACTAA**GAGCGCTG	
L. mirantina	TACGAT**CCAAT**CAGATTGAAATGTT**TTACTAA**TGGACACC	
L. dasiensis	GATGAG**CCAAT**TAGAGTGACTATGGCCTATGGAATCAATA	
L. meyersii	CGACAG**CGAAT**CCTAATATGATGTT**TAAGTCA**AAGCCATA	
L. nothofagi	CCGATG**CCAAT**AGGTCCTTCGTGTACAT**TTAGAAA**AGAAC	
L. kluyveri	CTGAAC**CCAAT**ACCCATAGGTCCTTCGTGTACGTTCAAGA	
L. lanzarotensis	ATTTTA**CCAAT**ATTAGCTGAACCAGGACTGGGGAAGAGTA	
K. lactis	TTACAA**CGAAT**CAGTCCCGCTGCGCGTTTCCTACTTGATC	
E. gossypii	CCTTAC**CCAAT**CGTAACATCCCTCAAAGGGACGAAGAATA	
C. fabianii	AGAGAT**CCAAT**TCGATAAAGACGAGTGTTGAAGTGTTTGA	HapX-like
M. farinosa	AAGGAA**CCAAT**TACACAAAAAGCAAGGATTATTATTACAC	
D. hansenii	AATAAC**CCAAT**ACCGATAATCGGCTAGGATTTGGAGTACC	
C. parapsilosis	AATTTT**CCAAT**CAAACTCCGCCCCCCCTCCCCCAGACTCC	
C. albicans	CCGAGA**CCAAT**AGAGCCTCAATCAATCTCGTATAATTAAT	
A. adeninivorans	TAATGT**CCAAT**CTAGCGGAGATACAGAACAAGACACACAG	

(B)

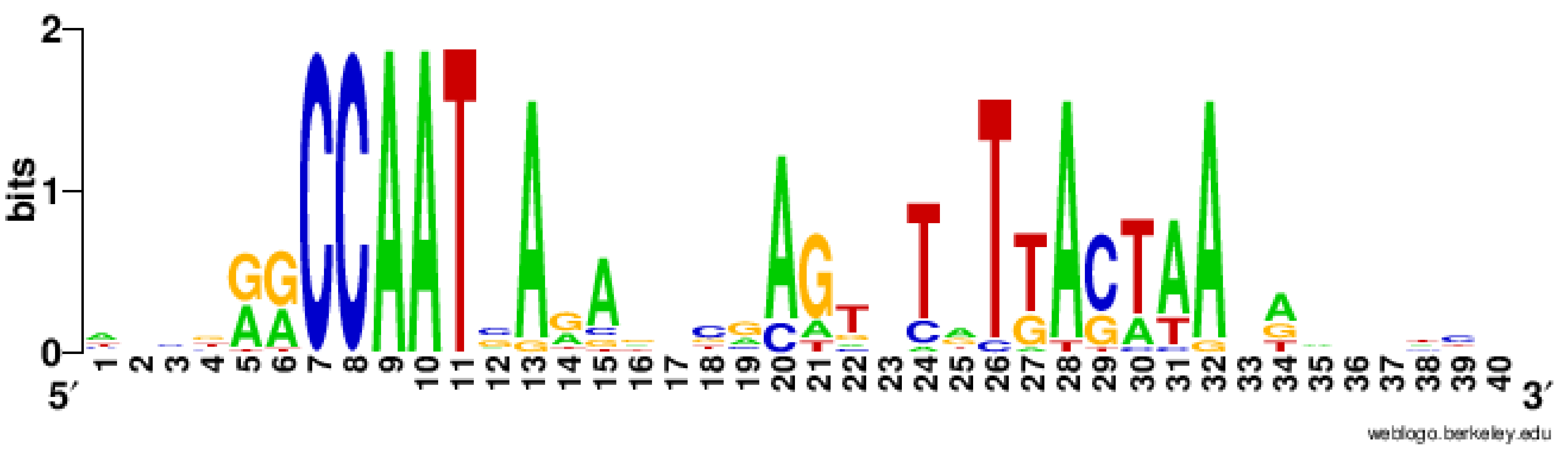

ISA1

Supplementary file S4: Conservation of the CCAAT-YRE bipartite motifs in *ISA1* orthologues from 33 Hemiascomycetes yeast species. (A): Alignment of the CCAAT-YRE bipartite motifs. The sequences were arbitrarily aligned based on the CCAAT box. All sequences are oriented 5'-3' from left to right. The arrow on top indicate the sense of transcription of *ISA1*, i.e. the arrow oriented to the left indicates that the CCAAT-YRE motif is on the minus strand. (B): Seqlogo of the CCAAT-YRE alignments in the sequences from the *Saccharomycetaceae* species ("Yap5-like" species) presented in panel (A), excluding *C. castelli, Z. rouxii, Z. bailii, L. dasiensis, L. kluyveri, L. lanzarotensis, K. lactis* and *E. gossypii*.

(A)

S. cerevisiae	TTCTGA**CCAAT**GAGAACCCAGTTTG**TTAGTAA**GTGCATTT	Yap5-like (*Saccharomycetaceae*)
S. mikatae	TTTCGA**CCAAT**GAGAACTCAGCTCG**TTAGTAA**GTGTATTA	
S. kudriazevii	TCTTGA**CCAAT**GAGACCATAGTTCG**TTAGTAA**GTGTATCA	
S. bayanus	TTTCAG**CCAAT**GAGATCGCTCTTTG**TTAGTAA**GTGCGTTA	
C. glabrata	AAAGGG**CCAAT**GATGAGGTACAATA**TTAGTAA**GCCAATGA	
N. delphensis	TTTTGA**CCAAT**CAGAGACGAACAAA**TTAGTAA**CATACCCA	
C. nivariensis	TTTTGA**CCAAT**CAGAATCGAATAAAG**TAGTAA**TTAAATAT	
C. bracarensis	ATGTGG**CCAAT**CAGAAGCGAGAATA**TTAGTAA**ATGCAGTG	
C. castelli	TCGCAG**CCAAT**TTCGTAGGGAAGGATTTAT**TATCTAA**TTT	
N. bacillisporus	CTATTC**CCAAT**TCTTACAATGTCCGTAGCAAATCCCCTTC	
K. naganishii	TGTATC**CGAAT**TCTCCAGGGGTTCAACATAACTTTGATAT	
T. blattae	GTTCAG**CCAAT**AAAATCTCCGGATTTCACTA**TTACTAA**TG	
N. castellii	AGCGAG**CCAAT**GAATATGAAGGGGG**TTAGTAA**GTTGTTTG	
Z. rouxii	AATCGA**CCAAT**GAGATAAAAGATTA**TTAGTAA**AGCTCTAG	
Z. bailii	TGTAAG**CCAAT**TAACAAACAAAGTG**TTAGTAA**GGGACTTT	
T. Microellipsoides	TCAGAA**CGAAT**TCGAACGCAACTTTTGCCTCCTGCAATAT	
L. fermentati	GAATGA**CCAAT**CAGAAATCATTTCA**TTACA**C**A**TTTCAAGT	
L. thermotolerans	AGAAAC**CCAAT**CATAGGTCCTGTCA**TTACAAA**TTAACGTC	
L. quebecensis	GAGAAT**CCAAT**CATAGATCGCGTCA**TTACAAA**TTAACGTC	
L. mirantina	CCATGA**CCAAT**GTCAAGGAGTAAACAGAACATGCGTGTTC	
L. dasiensis	TGAGAG**CCAAT**CACAGCTAGGCTCA**TTACAAA**TCAGTATC	
L. meyersii	ACATAG**CCAAT**CAGAGACCGGTTCA**TTACAAA**TCGGCCAT	
L. waltii	ATCAAA**CCAAT**CACAGTCTGTTTTA**TTACAAA**TCGATAGT	
L. nothofagi	GAGGAA**CCAAT**CACAGGCCGGTTCA**TTACAAA**TTGACATT	
L. kluyveri	TATCAA**CCAAT**CAGATTGAAGTTCA**TTAGTTA**GCTTGTAG	
L. lanzarotensis	AGCAAG**CCAAT**CAGAATCGGTGTCA**TTACA**C**A**TCGATGTT	
E. gossypii	TGGCGG**CCAAT**GTTTCTTTTTCCCACCGTTTCAGGGTTTT	
M. farinosa	AAGTAT**CCAAT**TTTTTGTATTATATTAACTGAGAAAGAAG	HapX-like
D. hansenii	AAGAAA**CGAAT**ATTCAGAATGGTTTCATTTTATTCTGTTG	
C. parapsilosis	AGTGAA**CCAAT**ATAGAATCAATAGCGTAACAGACACCAAT	
C. albicans	TGAATA**CCAAT**CCTCTCATCTCTAGATTGTTGTTGATGGT	
A. adeninivorans	GTAAAG**CCAAT**TTGGTAGCAAAGACAGACAGACAGTGACT	
Y. Lipolytica	TGCTGA**CGAAT**AGCGGTTTGAATTTTGTCATGAAAAATCT	

(B)

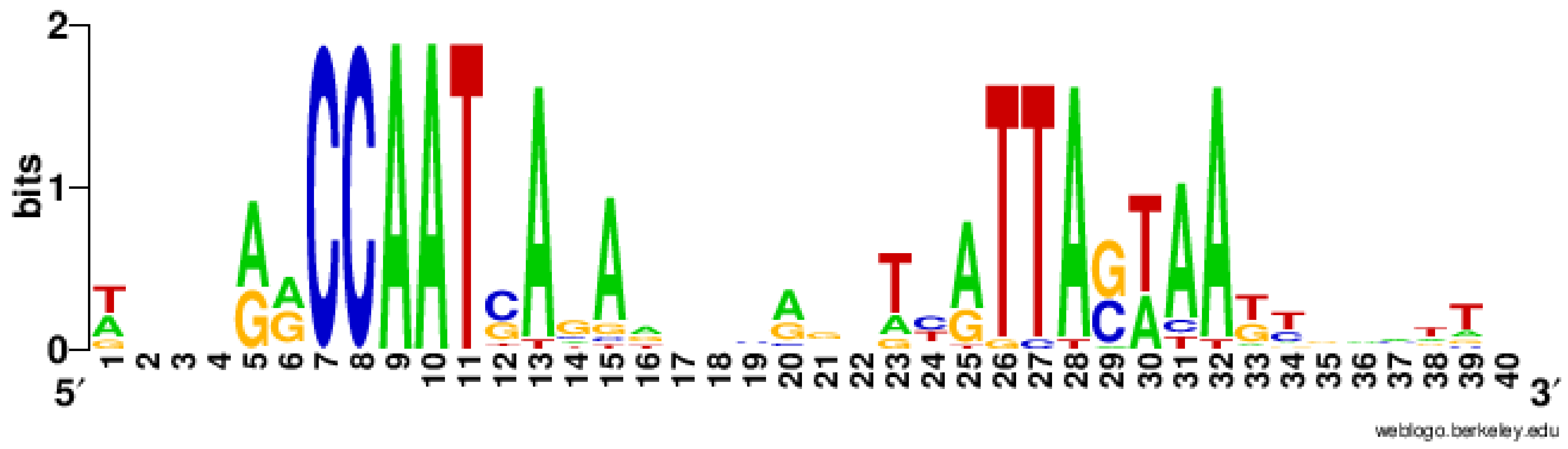

TYW1

Supplementary file S5: Conservation of the CCAAT-YRE bipartite motifs in *TYW1* orthologues from 33 Hemiascomycetes yeast species. (A): Alignment of the CCAAT-YRE bipartite motifs. The sequences were arbitrarily aligned based on the CCAAT box. All sequences are oriented 5'-3' from left to right. The arrow on top indicate the sense of transcription of *TYW1*, i.e. the arrow oriented to the left indicates that the CCAAT-YRE motif is on the minus strand. (B): Seqlogo of the CCAAT-YRE alignments in the sequences from the *Saccharomycetaceae* species ("Yap5-like" species) presented in panel (A), excluding *C. castelli, N. bacillisporus, K. naganishii, T. microellipsoides, L. mirantina* and *E. gossypii*.

(A)

Yap5-like *(Saccharomycetaceae)*	HEM3
S. cerevisiae	GTCTGA**CCAAT**AAGAAACCGA**TGAGTAA**GCAATGAAGGTC
S. mikatae	TTTTAA**CCAAT**AAGATAACGA**TGAGTAA**GCAATGAAGGCC
S. kudriazevii	TTTTGA**CCAAT**AAGAAAACGA**TGAGTAA**GCAACGTACGCC
S. bayanus	TCTTGA**CCAAT**AAGATAACGA**TGAGTAA**GCAAAGAAGGCC
C. glabrata-1	TCCCAG**CCAAT**CACACACGCA**TTACTAA**GAATGCACGCCC
C. glabrata-2	AACGAG**CCAAT**CAGATCAATCGGG**TTAGTAA**GCAATTTCC
N. delphensis	GAGCAA**CCAAT**CACCGTCCCGGGAACACGCATT**TTAGTAA**
C. nivariensis	GGACAA**CCAAT**CACCGTCCCGCACT**TTAGTAA**TTTGCCCA
C. bracarensis	TTCTGC**CCAAT**CTTTGAATATGTGTG**TTAGTAA**GCTCGAC
C. castelli	CCAGCT**CCAAT**CCTCTTGAACTATTTCAAGGTTGAATGAA
N. bacillisporus	ATTTAA**TTAGTAA**TGGACTT**CGAAT**AAAACTTACTACACC
K. naganishii	CCGCTG**CGAAT**GAAACGAAGAGGAAAAAATGACGCTCTGG
T. blattae	CGTGGC**CCAAT**GATGATGTGTAAAG**TGAGTAA**TGTGCAGA
N. castellii	GACTTT**CCAAT**TTATTCAAGTTTGCATATTTGGATGGTTT
Z. rouxii	TAACTA**CGAAT**TTTTATAGAA**TTACAAA**TTAATTCGAATG
Z. bailii	TGTTCC**CGAAT**CAGGAACGGAGATCCTTGAGGGATTCCCT
T. Microellipsoides	CATCGA**CGAAT**TATATCAGACTTATTTAACTC**TTAGTAA**T
L. fermentati	GCGATT**CCAAT**TGTATGAATACATCACTGTAAATATCATT
L. meyersii	GTGCCG**CCAAT**AAGCTCGAGCTTTGAAAGACACTTTTATT
L. mirantina	TGGTAT**CCAAT**CCGCTTTCCCCTTGCGTGAATAAGTGATA
L. dasiensis	CGCTAG**CCAAT**TGAGGTCCCAGAAGCACTCTACCCAGCGG
L. cidri	AGGTCA**CCAAT**ACTGCTATCCATTTGTCAATTCACAACGA
L. waltii	ATTTGT**CCAAT**GCGCTGCACTGAATTGGATGAGACTTCGC
L. nothofagi	GGAAAT**CCAAT**CTGCCCTCATCCTGCCAGTAATCGCCACA
L. kluyveri	AATTTT**CCAAT**TTTTTTCCTAGTTATCTTTTCTATCAAAA
K. lactis	TCTCGA**CCAAT**AAACACTCCTTCCAGTTTTCGGCCGCTTA
E. gossypii	ACTGAA**CCAAT**TCTTATGTGGTATATACTATATCATTAAT

Supplementary file S6: Conservation of the CCAAT-YRE bipartite motifs in *HEM3* orthologues from 27 Hemiascomycetes yeast species. (A): Alignment of the CCAAT-YRE bipartite motifs. The sequences were arbitrarily aligned based on the CCAAT box. All sequences are oriented 5’-3’ from left to right. The arrow on top indicate the sense of transcription of *HEM3*, i.e. the arrow oriented to the right indicates that the CCAAT-YRE motif is on the plus (coding) strand.

(A)

	→	
S. cerevisiae	TCAGCG**CGAAT**TCCACCTAGCCTTCTGCAAAAGTTCTTAA	
S. mikatae	TCGTCA**CCAAT**GATCAAGCTCCAGTAAAATTCGAGAAGAG	
S. kudriazevii	AAGAAG**CCAAT**TCCAACGGGAAGAATCGTGATACCGTCCT	
S. bayanus	TCGTAA**CCAAT**GATCAAACTTTACCAAGTGCGAAAGAATG	
C. glabrata	TATGAA**CCAAT**AGGATGCCC**TTACTAA**GTGTGGGGACTTC	
N. delphensis	TCCCAT**CCAAT**GATATCCTCGTATA**TTACTAA**TGGGGGGG	
C. nivariensis	CCTCAG**CCAAT**AATATTAGAGTATC**TTAGTAA**TCTGGTAA	
C. bracarensis	GACGGG**CCAAT**GATATCATAGAATG**TTAGTAA**TTGGTTTA	
C. castelli	ACAGAG**CCAAT**TGGCGAAAAGATGTCGTAGAGGAGGGCTT	
N. bacillisporus	GACATT**CGAAT**TCGTTAGCATCACACACATCTTCTTAGAG	
K. naganishii	CATCTC**CCAAT**TAGGAATAAAACCCACGAAATTTACGGCA	
T. blattae	AGGCAA**CCAAT**TAAAGACCTACTGTTATTAATGAAGTTAT	
	←	
N. castellii	GGAGGG**CCAAT**GAGAACAGAGAATG**TTACTAA**CACTCTTG	
	→	
Z. rouxii	ATCATT**CCAAT**CACTTGTAACCATTGGAAGGAG**TGACTAA**	Yap5-like
Z. bailii	GTTTTC**CCAAT**CCTCAATCCTTTTATACTAAAGAGTCCCT	*(Saccharomycetaceae)*
T. Microellipsoides	TTACTT**CCAAT**CCTATTTCTATCCACTCATGTTCACGATC	
L. fermentati	CCAGGT**CGAAT**AGTCACTTTAACCGGTATGATAAGCTAAT	
L. thermotolerans	ACTTTT**CCAAT**ACAAGACTTATAAGTTGTAGTTTCAGGCG	
L. lanzarotensis	TGCTCA**CCAAT**CGCTCCTCAGAAACCTTTTGTGCGAACAT	
L. dasiensis	TTCCAT**CCAAT**ACCATGCGCGCTGAAACTCACAATACGAT	
L. cidri	TTTTAT**CGAAT**GCAAAACTCATCGGCACCAGCGATCAAGC	
L. waltii	CAAAAA**CCAAT**TTACACGTCTGATCAATAATTGAAACATG	
L. nothofagi	CGACTA**CCAAT**TACCAGTAAGCTCTACACACGCAAGACTT	RLI1
L. kluyveri	TTTTGG**CCAAT**AAAAGACACAAAAAATATTTCTTGGCTGG	
K. lactis	AAAAAA**CGAAT**ACCACTACTAAAGAATCAGAACCTATTAC	
E. gossypii	TGGGTT**CGAAT**CATTATACAGAAGGCCATCTCATAGGTAT	

Supplementary file S7: Conservation of the CCAAT-YRE bipartite motifs in *RLI1* orthologues from 26 Hemiascomycetes yeast species. (A): Alignment of the CCAAT-YRE bipartite motifs. The sequences were arbitrarily aligned based on the CCAAT box. All sequences are oriented 5'-3' from left to right. The arrow on top indicate the sense of transcription of *RLI1*, i.e. an arrow oriented to the right indicates that the CCAAT-YRE motif is on the plus (coding) strand, an arrow oriented to the left indicates that the CCAAT-YRE motif is on the minus (non coding) strand.

(A)

S. cerevisiae	AAAAATGTGCAAAAGCTTATCGGAG**CCAAT**CGGATTGACG
S. mikatae	ATATATGTCCGAGGCCTTATCGGAG**CCAAT**CGGATCAATG
S. kudriazevii	ATTGATTAATTTGGTGAAGAGAAAG**CCAAT**AATAGGACAA
S. bayanus	ATATGATCCGTCGGGCTTATCAGAG**CCAAT**CGGACGGCCG
C. glabrata	ACCATT**TTAGTAA**TACCGGTATTGT**CCAAT**AGATAAGGAA
N. delphensis	AAACTCCGATTGTATAAAAGACGTG**CCAAT**GGACGTATTT
C. nivariensis	TAGAAA**TTAGTAA**AAACTTAGTA**CAAT**TTCTAATTCTACA
C. bracarensis	AATTAA**CCAAT**AATTCAAAC**TTAGTAA**GTTCTTATCAGTA
C. castelli	GTACCA**CGAAT**CAATCTCTGCACTGTATGCTGTGGATAAA
K. naganishii	GGGGGT**CCAAT**CATATCCCGGGCGCGTTATCTTTCACGTG
T. blattae	GTAATT**CCAAT**CTCCGTGCTTGCAGATCATATCTCATCTA
N. castellii	AACTGA**CCAAT**CAAATCAAGGAAGG**TTACTAA**CTTTTTGA
Z. rouxii	TAATAC**CCAAT**CAATCTTTAATTGTTAAAAAGAGAATGTG
Z. bailii	GGAGCC**TTAGAAA**CTTTATTTTTTAG**CGAAT**TCATGTAGA
T. Microellipsoides	TAAATC**CCAAT**CTACTACAATCG**TTACAAA**TCGGTTTATC
L. fermentati	TGTGGG**CCAAT**CGGAGCGGCGCCGCTCAGACGGGGGCGCG
L. thermotolerans	CGCCAA**CCAAT**CAGACACGGTTCCC**TTAGA**T**A**GTAGCGCA
L. cidri	CGTGGG**CCAAT**AGAAAAACGAATCC**TTAGAAA**CATCAGGA
L. lanzarotensis	AATGCA**CCAAT**CAGAGGTCCGGCCCTCTTAAAACGTCGCA
L. dasiensis	TTTAGG**CCAAT**CATATTCCAGAATCTCAGAAAACAGCGTA
L. quebecensis	CGTCAA**CCAAT**CAGATGCGGCCACCTTAGATAATAGCGCA
L. nothofagi	TTTCTA**CCAAT**CAGATGGCCGCACCCTAGAGAACCGCGTA
L. kluyveri	TCTCCATATCTGTCCCAGCCGCTCG**CCAAT**CCCGTGCTCA
K. lactis	ATCGGA**CCAAT**CATATCGAGGTTGCTCGAATTTGCTGGCA
E. gossypii	CTTCGG**CCAAT**CAGAAGCCTTCTACGGTGCGCCGCCCGCA

Yap5-like
(Saccharomycetaceae)

GLT1

Supplementary file S8: Conservation of the CCAAT-YRE bipartite motifs in *GLT1* orthologues from 25 Hemiascomycetes yeast species. (A): Alignment of the CCAAT-YRE bipartite motifs. The sequences were arbitrarily aligned based on the CCAAT box. All sequences are oriented 5'-3' from left to right. The arrow on top indicate the sense of transcription of *GLT1*, i.e. an arrow oriented to the right indicates that the CCAAT-YRE motif is on the plus (coding) strand, an arrow oriented to the left indicates that the CCAAT-YRE motif is on the minus (non coding) strand.

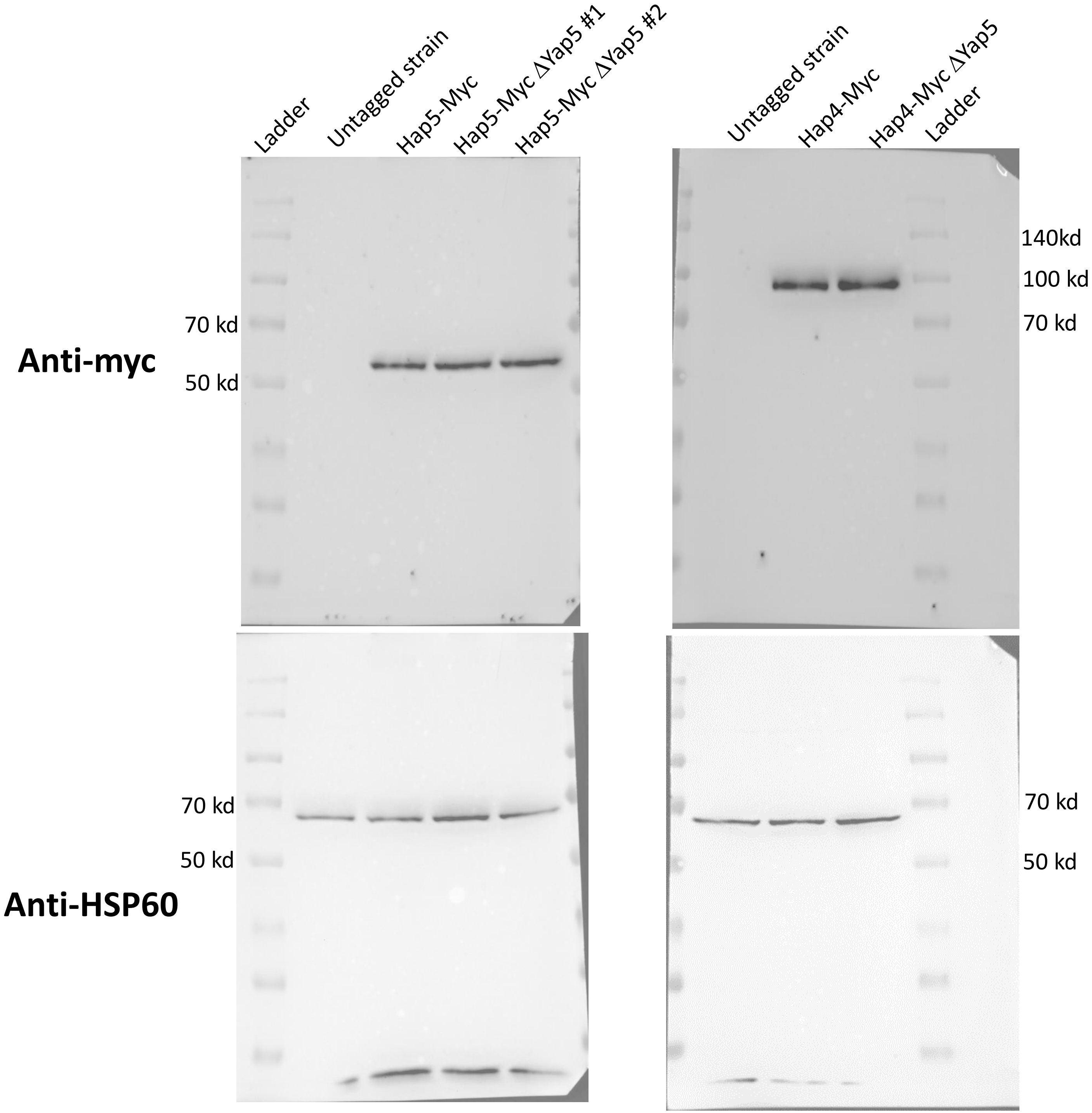

Supplementary file S9: Western blot analyses of the Hap5-myc (left panels) and Hap4-myc (right panels) in presence or absence of *YAP5*. The western blots were performed as previously described (Thiébaut et al., 2017). The myc-tagged proteins (upper panels) were detected using the 9E10 anti-myc antibody from Roche. Hsp60 was used as a loading control (lower panels). The anti-Hsp60 antibody is a gift from Jean-Paul Di Rago. Two independant clones of the Hap5myc Δyap5 background are shown. The untagged parental strain was used as a negative control for the anti-myc signal.

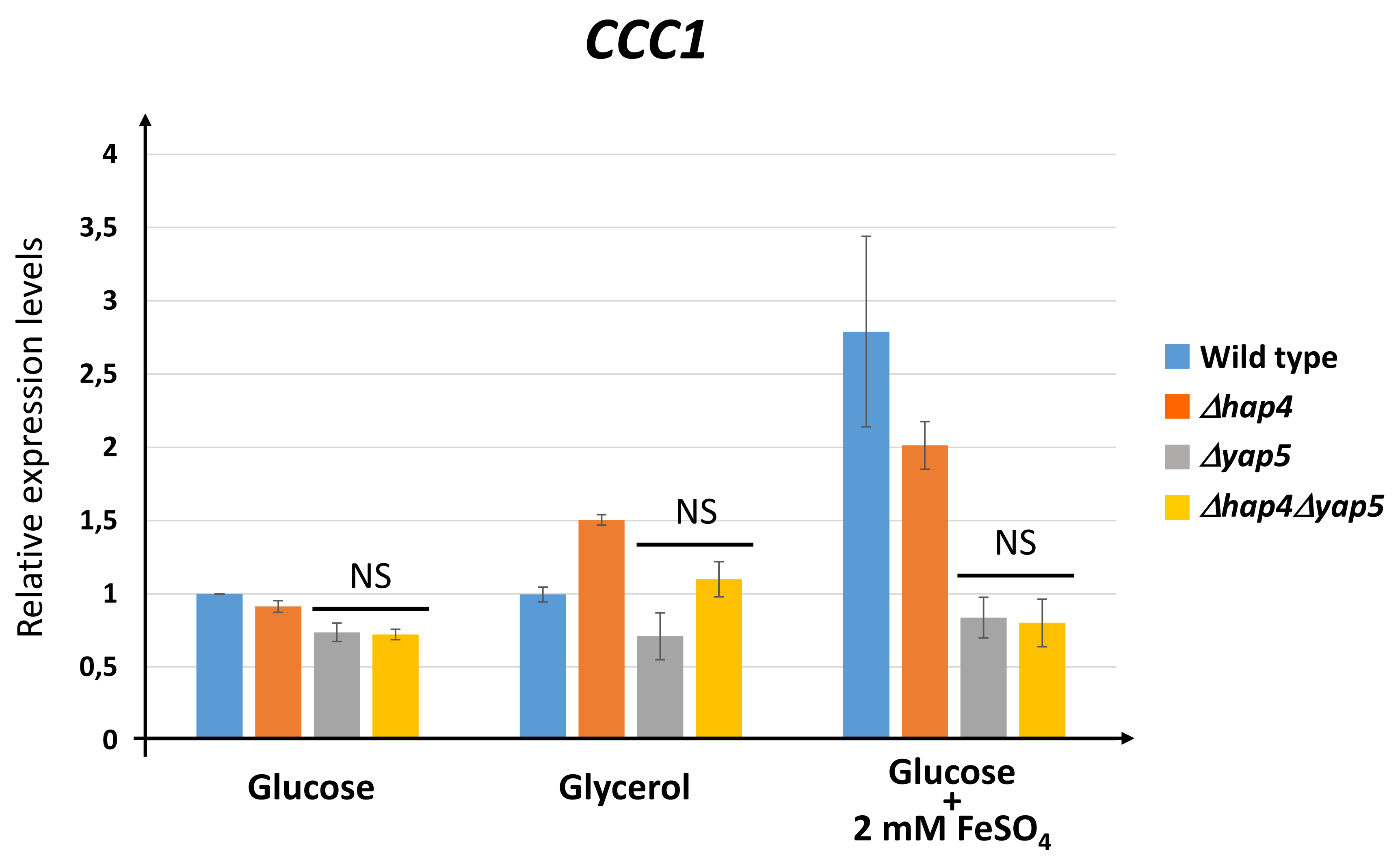

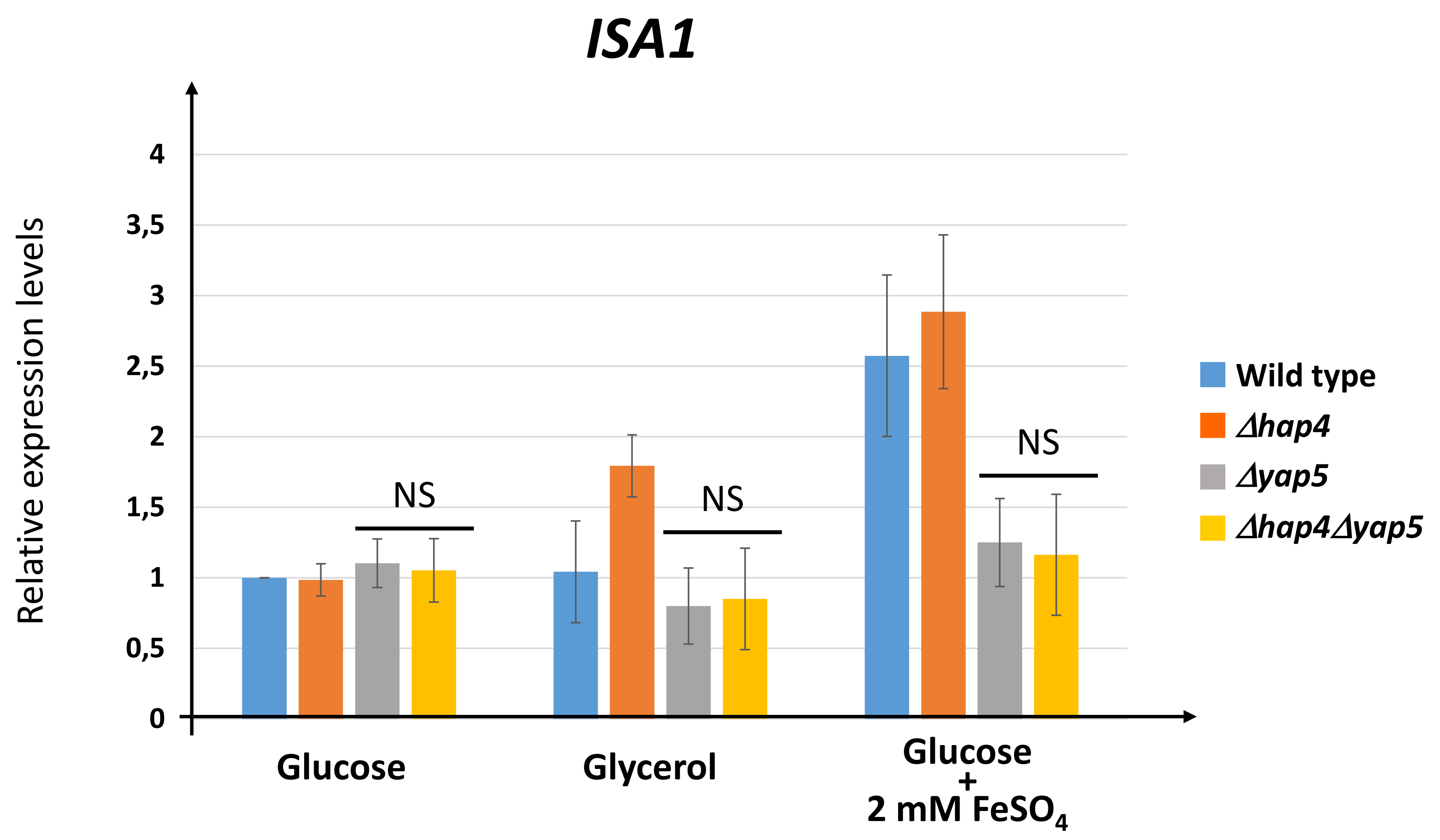

Supplementary file S10: Analyses of the impact of a double deletion of *HAP4* and *YAP5* on the expression of *CCC1* (upper histogram) and *ISA1* (lower histogram). The relative expression levels were measured by RT-qPCR in wild type, *Δhap4*, *Δyap5* and *Δhap4Δyap5* strains grown in glucose, glycerol or glucose + iron excess (2 mM $FeSO_4$). The values represent the expression levels of the *CCC1* or *ISA1* genes relative to *ACT1* (used as an internal control) and to the wild type grown in glucose, which was arbitrarily set to 1. The experiments were performed six times on biologically independent samples, except for glycerol which was based on three replicates only. Error bars represent the pearson standard deviation. The results of a t-test comparing the expression levels of *CCC1* and *ISA1* between the *Δyap5* and the *Δyap5Δhap4* strains are symbolized as follows: NS: $p > 0.1$.
